# Prins cyclization-mediated stereoselective synthesis of tetrahydropyrans and dihydropyrans: an inspection of twenty years

**DOI:** 10.3762/bjoc.17.77

**Published:** 2021-04-29

**Authors:** Asha Budakoti, Pradip Kumar Mondal, Prachi Verma, Jagadish Khamrai

**Affiliations:** 1 Division of Molecular Synthesis & Drug Discovery, Centre of Biomedical Research (CBMR), SGPGIMS Campus Raebareli Road, Lucknow, 226014 Uttar Pradesh, India

**Keywords:** asymmetric, natural products, Prins cyclization, stereoselective, tetrahydropyran

## Abstract

Functionalized tetrahydropyran (THP) rings are important building blocks and ubiquitous scaffolds in many natural products and active pharmaceutical ingredients (API). Among various established methods, the Prins reaction has emerged as a powerful technique in the stereoselective synthesis of the tetrahydropyran skeleton with various substituents, and the strategy has further been successfully applied in the total synthesis of bioactive macrocycles and related natural products. In this context, hundreds of valuable contributions have already been made in this area, and the present review is intended to provide the systematic assortment of diverse Prins cyclization strategies, covering the literature reports of the last twenty years (from 2000 to 2019), with an aim to give an overview on exciting advancements in this area and designing new strategies for the total synthesis of related natural products.

## Introduction

**Scheme 1:**
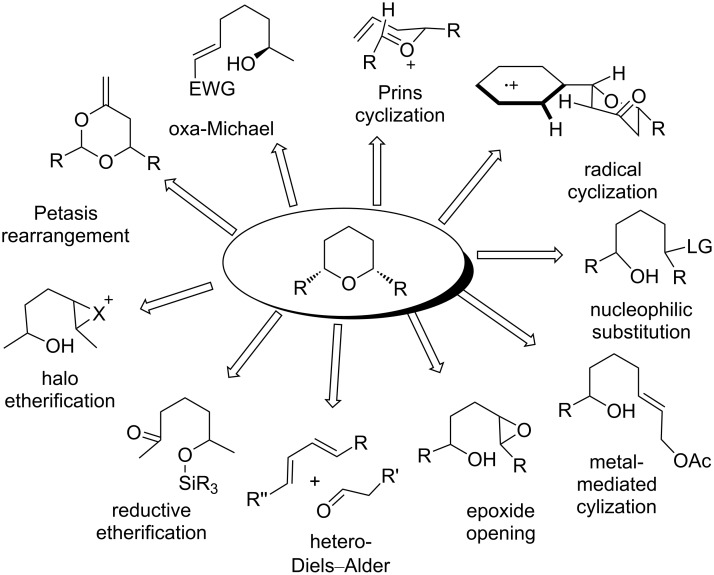
General strategy for the synthesis of THPs.

6-Membered saturated oxygen heterocycles, known as tetrahydropyran (THP), are recognized as privileged scaffolds, present in a variety of biologically important natural products, such as polyether antibiotics, marine toxins, pheromones, and pharmaceutical agents. These structural motifs are frequently used as synthons and as key intermediates for natural product synthesis. Therefore, the development of stereoselective synthetic methods for the substituted THP subunit has long been the area of fundamental research in organic chemistry. Thus far, several methods have been devised for the construction of substituted tetrahydropyran rings. Since the year 2000, a number of conceptually different reactions have been developed for the efficient construction of THP rings and were eventually employed in the total synthesis of natural products [1–8]. Prins and related cyclization reactions [9–10], hetero-Diels–Alder cyclization [11], cyclization onto epoxides [12], Petasis–Ferrier rearrangement [13], intramolecular oxa-Michael reactions [14], cyclization through oxidative C–H bond functionalization [15], ring-closing metathesis (RCM) [16], halo etherification [17], reductive etherification [18–19], and metal-mediated cyclization [20–21], etc. are the most frequent strategies utilized for THP ring construction (Scheme 1). Amongst all, the Prins reaction has proven as a powerful technique in the stereoselective synthesis of the THP key scaffold and its application towards the total synthesis of natural products. Many advancements were also taking place in Prins cyclization methodologies over that period of time. This appraisal aims to bring together the work of many research groups in the area of the development of Prins and related cyclization strategies along with the discussion on the general mechanistic part. We sincerely hope that this review will deliver a snapshot of the up-to-date state of the inventiveness in this field, and most importantly, it will give an inspiration to the reader to take up the challenge and contribute greater advances in this area in the future. This review comprises the literature reports over the last twenty years and its advances. It is likely that some references may have escaped our attention unintentionally, for which we would greatly apologize to those whose contribution in this area has not been included.

## Review

### Prins cyclization: general

**Scheme 2:**
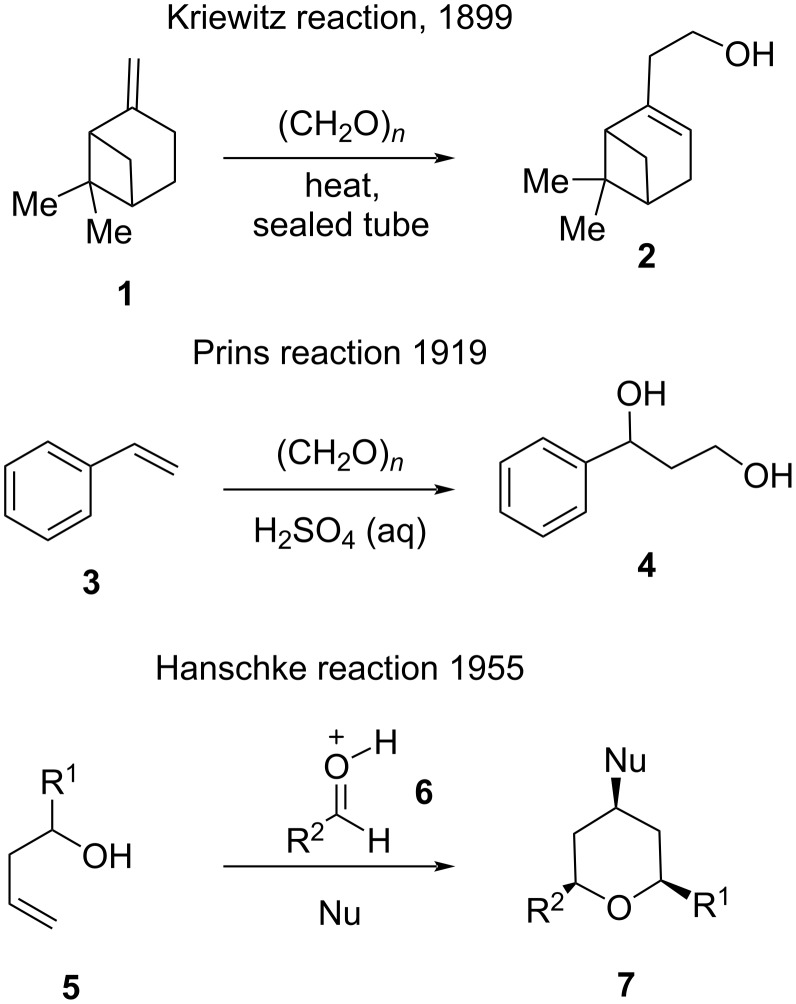
Developments towards the Prins cyclization.

For the first time, in 1899, Kriewitz [22] reported the synthesis of unsaturated pinene alcohol through a thermal ene reaction using β-pinene and paraformaldehyde. After nearly twenty years, Prins explored this reaction further for the synthesis of diol by the condensation of styrene and paraformaldehyde in the presence of a Brønsted acid [23–24]. The major breakthrough for this reaction was reported by Hanschke in 1955, when the THP ring was selectively constructed through a Prins reaction involving 3-butene-1-ol and a variety of aldehydes or ketones in the presence of acid (Scheme 2) [25].

**Scheme 3:**
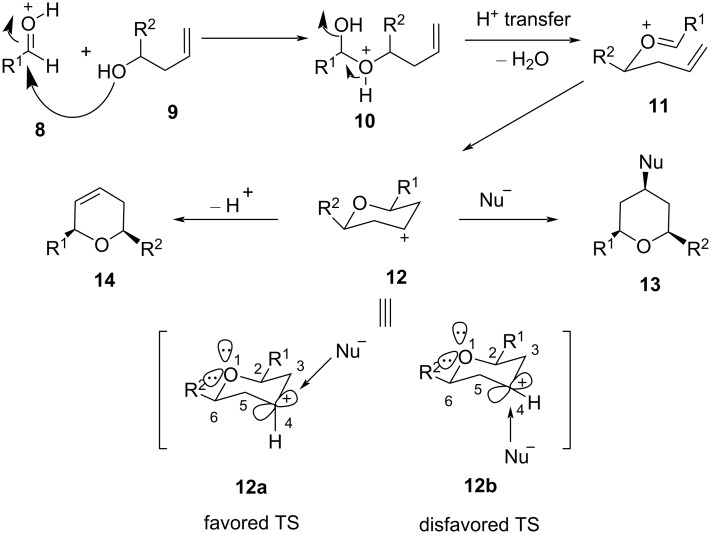
General stereochemical outcome of the Prins cyclization.

Although the Kriewitz reaction was an ene reaction, the mechanism of the reaction was described to proceed via an oxocarbenium ion intermediate captured by a π-nucleophile, followed by the addition of an external nucleophile, leading to the formation of products. Since then, the Prins cyclization emerged as the most commonly used strategy for the stereoselective construction of the THP ring, and its application lead to some excellent reviews on the Prins reaction [26–27]. In general, an *endo* cyclization proceeds via an oxocarbenium ion intermediate in a stereoselective manner for THP ring formation as shown in Scheme 3.

**Scheme 4:**
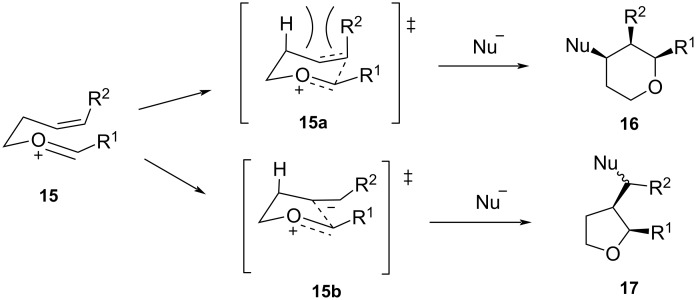
Regioselectivity in the Prins cyclization.

The outcome of exclusive *cis-*stereoselectivity in the Prins cyclization might be attributed to the most favorable conformation adopted by **12** with equatorial orientation of the 2,6-substituents (R^1^ and R^2^). Alder and co-workers explained the formation of all-*cis-*2,4,6-trisubstituted THPs with the help of density functional theory (DFT) and stated that in the presence of an external nucleophile, the stabilization of the carbocation intermediate is favored through hyperconjugation [28]. The vacant p-orbital of C4 in TS **12a** overlaps efficiently with the HOMO of the incoming nucleophile in an equatorial attack. Furthermore, this pseudoaxial C4 hydrogen atom in TS **12a** leads to an optimal overlap between σ and σ* of C2–C3 and C5–C6 with the coplanar equatorial lone pair of the oxygen atom and the empty p-orbital at C4. These orbital stabilizations, along with the lack of 1,3-diaxial interaction experienced by the incoming nucleophile (mostly halide) leads to the preferential equatorial attack over an axial attack by the nucleophile (Scheme 3) to give all-*cis*-2,4,6-trisubstituted THPs. In the absence of an external nucleophile, the successive proton loss leads to the formation of the 2,6-disubstituted dihydropyran. The regioselectivity of the Prins reaction is explained through the intermediates formed during the course of the reaction (Scheme 4). The *Z*-homoallylic alcohol reacts with an activated aldehyde to give oxocarbenium ion **15**, wherefrom two competing transition states, **15a** and **15b**, can possibly form. In the 6-membered chair-like transition state **15a**, there is a 1,3-diaxial interaction between “H” and the substituent R^2^, while for the other five-membered transition state **15b**, there is no such 1,3-diaxial interaction, which favors the formation of tetrahydrofuran product **17** instead of the tetrahydropyran **16** (Scheme 4).

**Scheme 5:**
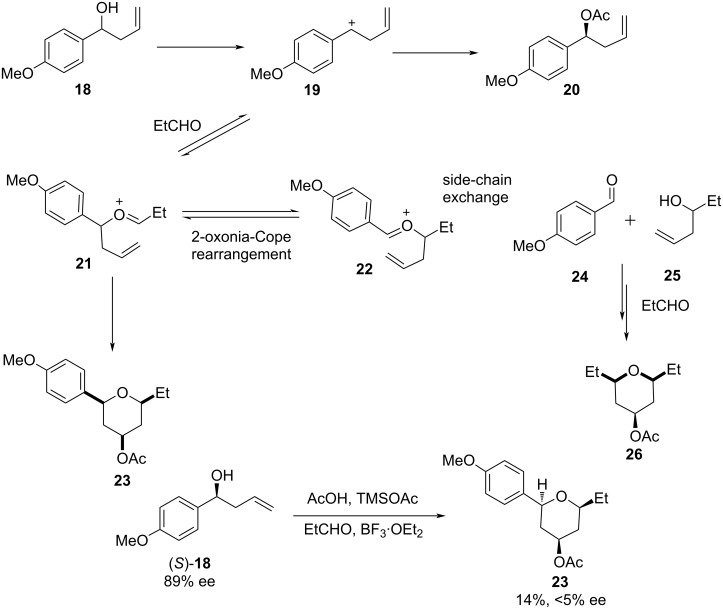
Mechanism of the oxonia-Cope reaction in the Prins cyclization.

Although the Prins cyclization is one of the powerful tools for the construction of 2,6-disubstituted THPs, there are some limitations that restrict a wide applicability. The major drawbacks identified with the Prins cyclization are the racemization due to competing oxonia-Cope rearrangement and side-chain exchange. Willis and co-workers studied the reactivity of the Prins reaction of different aryl group-substituted homoallylic alcohols **18** with propanal in the presence of a Lewis acid, which furnished the expected tetrahydropyran **23** as a single diastereomer via an oxocarbenium intermediate **21** (Scheme 5) [29–30].

**Scheme 6:**
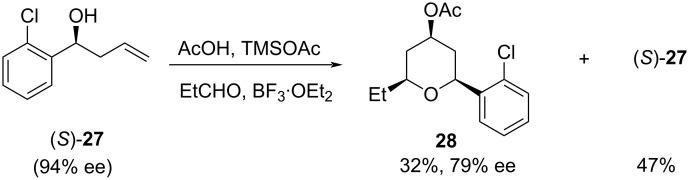
Cyclization of electron-deficient enantioenriched alcohol **27**.

The reaction was dependent from the nature of the aromatic ring, which plays a crucial role in the product formation. Homoallylic alcohols with an electron-rich substituent at the arene ring produced predominantly symmetric THP product **26** over the desired trisubstituted heterocycle **23**. The mechanism of the reaction was further investigated using enantioenriched homoallylic alcohol (*S*)-**18** with 89% ee, which favored 2-oxonia-Cope rearrangement to give THP **23** only in 14% yield and <5% ee. The poor enantiomeric excess of the product **23** indicates that the racemization takes place during the course of the reaction. It was explained that the reason for the loss of optical purity was due to the formation of a benzylic cation, which is stabilized by the electron-rich aromatic substituent. In contrast, the reaction with aromatic aldehydes equipped with the electron-deficient substituent produced the desired trisubstituted THP along with recovered starting material. The enantioenriched homoallylic alcohol bearing an electron-deficient substituent, **27** (94% ee), was investigated with propanal, which proceeded with high selectivity to give the corresponding THP **28** (79% ee, 32% yield) along with some recovered starting material (47%), as shown in Scheme 6.

**Scheme 7:**
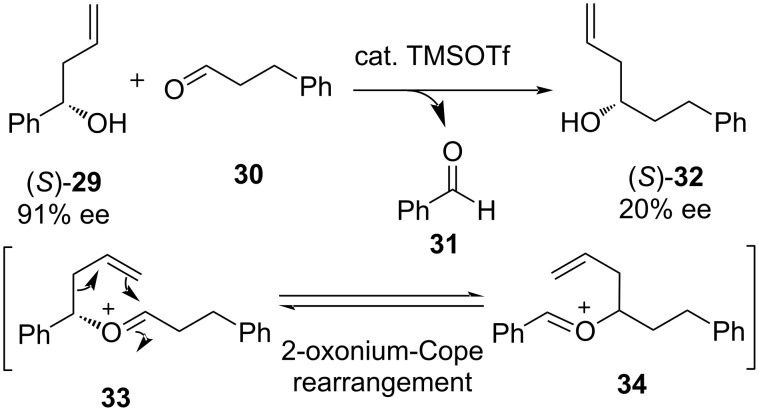
Partial racemization through 2-oxonia-Cope allyl transfer.

Partial racemization was also reported at the same time by reversible 2-oxonia-Cope rearrangement and via side-chain exchange [31–33]. The racemization occurs during allyl transfer as a result of 2-oxonia-Cope rearrangement through a 3,3-sigmatropic shift, which plays a crucial role during the reaction, as shown in Scheme 7.

**Scheme 8:**
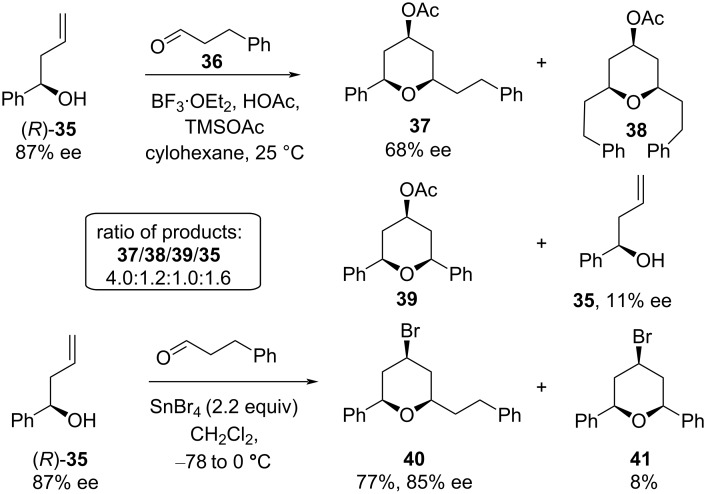
Partial racemization by reversible 2-oxonia-Cope rearrangement.

The Prins cyclization between alcohol (*R*)*-***35** and aldehyde **36** was investigated under different Lewis acid conditions, as shown in Scheme 8 [33]. Cyclization promoted by BF_3_·OEt_2_/HOAc led to partial racemization of the desired product **37** (from 87% ee to 68% ee) and formation of side-chain exchange products **38** and **39** (symmetric tetrahydropyran). Presumably, this observation stands in support of the intervention of a 2-oxonia-Cope-mediated side-chain exchange reaction and is entirely consistent with Willis and co-workers’ result [29], which leads to the partial racemization observed in the desired product formation. Another Lewis acid, SnBr_4_, was found to be more efficient than BF_3_·OEt_2_/HOAc in terms of retention of enantiopurity in major product **37** during cyclization (from 87% ee to 85% ee, Scheme 8). This could probably be due to a faster rate of cyclization with SnBr_4_, which suppressed the competing 2-oxonia-Cope process.

**Scheme 9:**
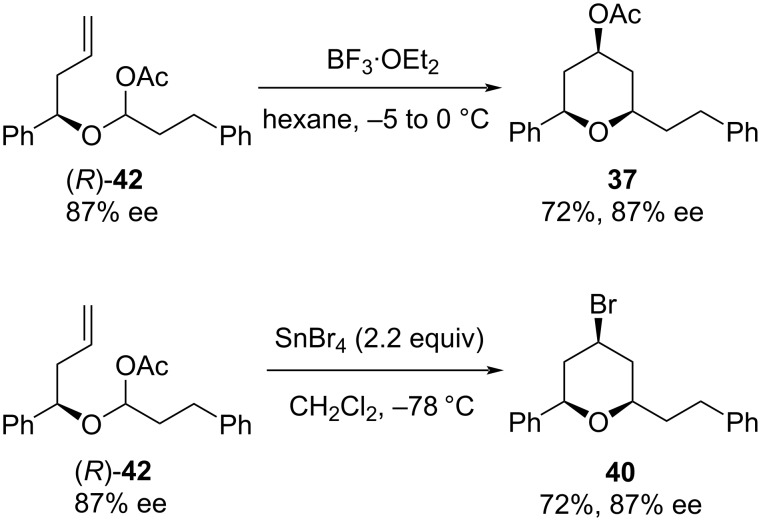
Rychnovsky modification of the Prins cyclization.

In order to stop racemization during the Prins cyclization, a substrate in which an oxocarbenium ion is generated from a masked aldehyde bearing a homoallylic alcohol moiety has been examined. In this context, the α-acetoxy ethers with different functionalities at C4 were examined in the presence of a variety of Lewis acids, and it was found that the α-acetoxy ether (*R*)-**42** underwent Prins-type cyclization in the presence of BF_3_·OEt_2_ as well as SnBr_4_ to deliver the desired **37** and **40**, respectively, without loss of optical purity (Scheme 9) [34–35].

**Scheme 10:**
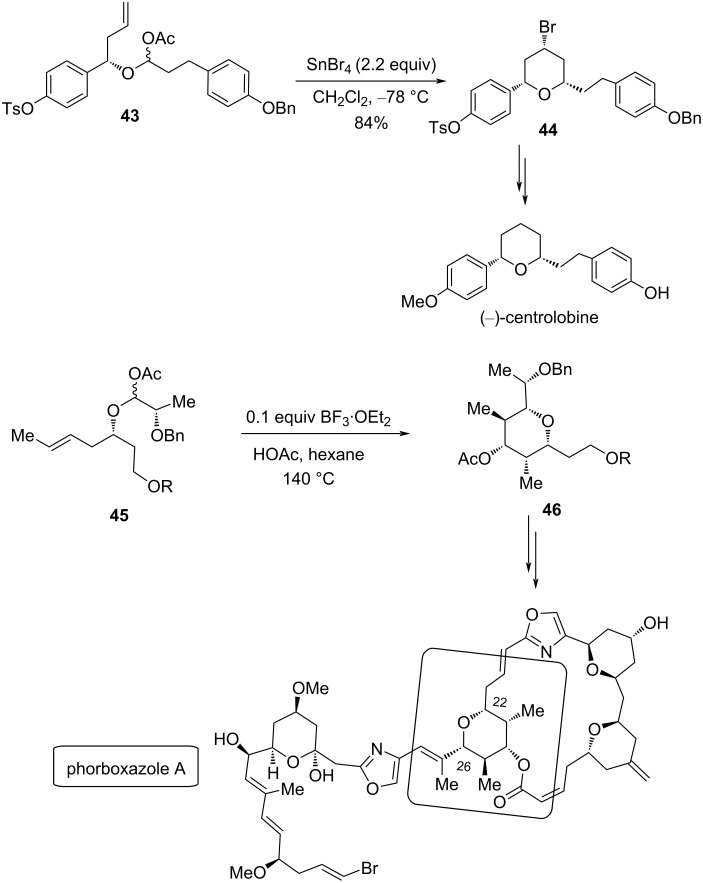
Synthesis of (−)-centrolobine and the C22–C26 unit of phorboxazole A.

This strategy was successfully utilized for the synthesis of the natural product (−)-centrolobine [33] and for the stereoselective synthesis of the C20–C27 tetrahydropyran segment of phorboxazole A (Scheme 10) [36].

### Axial selectivity in the Prins cyclization

**Scheme 11:**
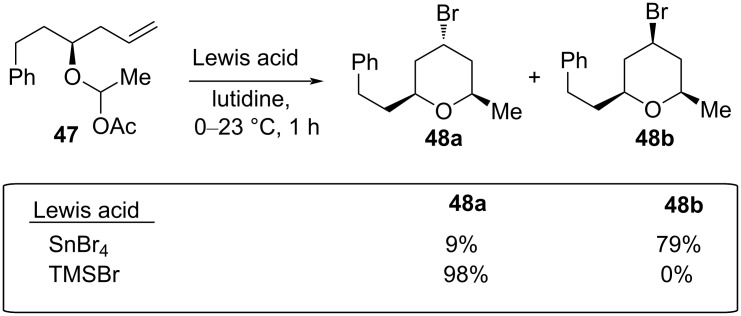
Axially selective Prins cyclization by Rychnovsky et al.

To overcome the racemization process, the axially selective Prins cyclization was explored with a variety of substrates, which produced the corresponding THPs in excellent selectivity and good to excellent yield [37]. The experimental modification under segment coupling gave entirely the 4-axial product. For example, treatment of **47** with SnBr_4_ produced axial and equatorial products **48a** and **48b** in a 9:79 ratio under typical segment coupling. This selectivity was further improved for the formation of **48a** by exclusively using TMSBr as a Lewis acid, as shown in Scheme 11.

**Scheme 12:**
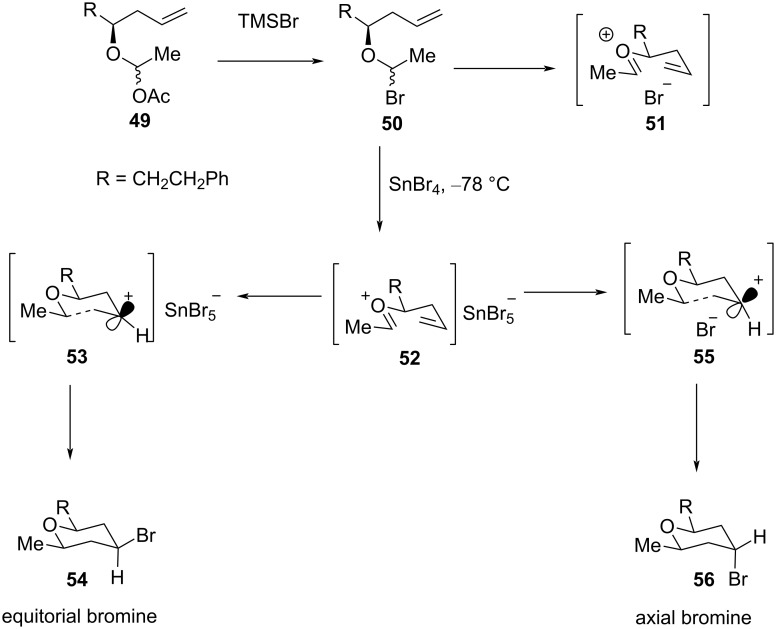
Mechanism for the axially selectivity Prins cyclization.

The mechanistic rationale for an axially selective Prins cyclization is explained in Scheme 12 [38]. It is proposed that the reaction of **49** with TMSBr forms an intermediate **50**, which, after solvolysis, affords an intimate ion pair **51**. The proximal addition of a bromide ion to **51** produces axial adduct **56** exclusively. However, when SnBr_4_ is used as a Lewis acid, oxocarbenium ion **52** is formed via **50**. The counterion SnBr_4_^−^ being much less nucleophilic than the Br^−^ ion allows the formation of a solvent-separated ion pair **53**, which results in the bromide addition to **53** preferentially from an equatorial position (Scheme 12).

### Mukaiyama aldol–Prins cyclization

**Scheme 13:**
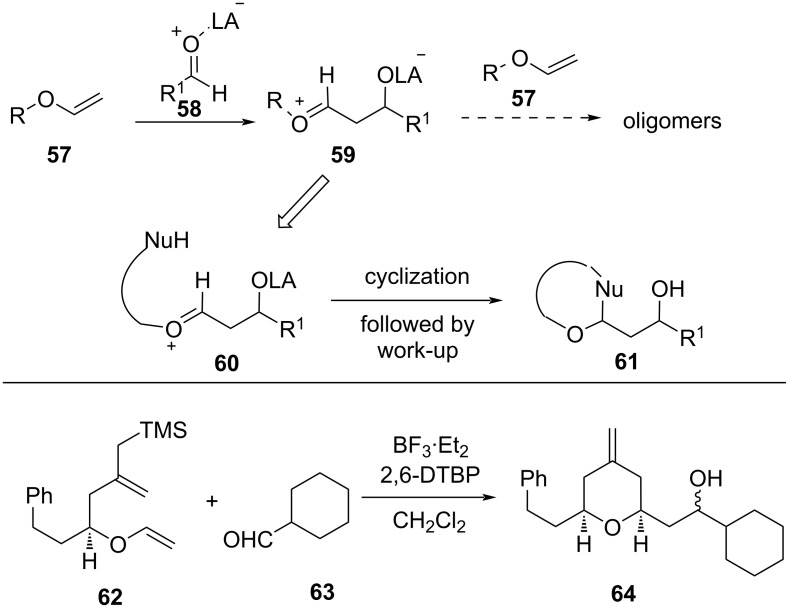
Mukaiyama aldol–Prins cyclization reaction.

The Mukaiyama aldol–Prins (MAP) cyclization has also been explored for the synthesis of tetrahydropyran. In this approach, the side reaction is avoided by introducing a nucleophile into the enol ether, which traps the reactive oxocarbenium ion intermediate **60**, leading to the formation of THP [39]. The first example of an MAP cascade reaction was reported by Rychnovsky and co-workers using allylsilane **62** as an internal nucleophile, as shown in Scheme 13 [40].

**Scheme 14:**
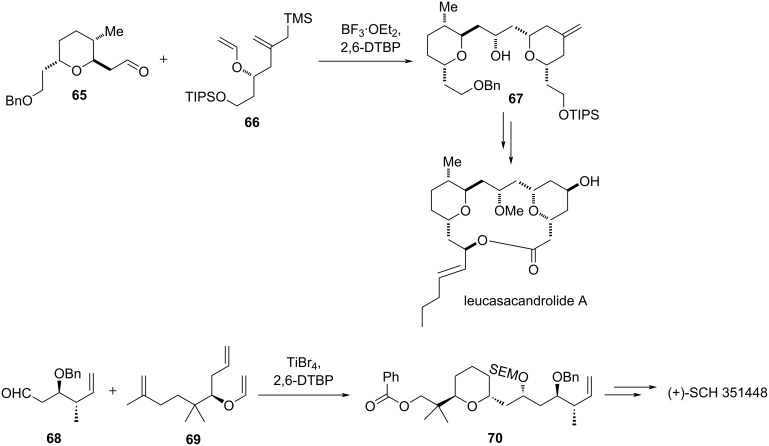
Application of the aldol–Prins reaction.

This approach was further extended to the synthesis of the macrolide lecasacandrolide A [41]. BF_3_·OEt_2_ in combination with 2,6-di-*tert*-butylpyridine (DTBP) was a suitable combination for the synthesis of the THP unit of leucasacandrolide A, while TiBr_4_ [42] was found suitable in conjunction with DTBP for the synthesis of polyketide SCH 351448 [43], as shown in Scheme 14.

**Scheme 15:**
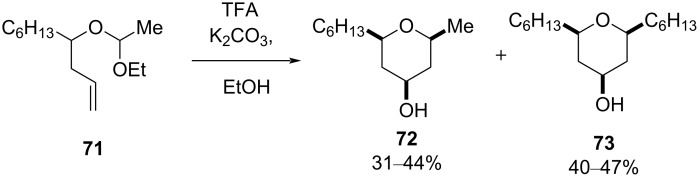
Hart and Bennet's acid-promoted Prins cyclization.

Hart and Bennett have also examined the trifluroacetic acid-catalyzed Prins cyclization of acetal **71** to afford **72** along with side-chain-exchanged product **73** (Scheme 15) [44].

**Scheme 16:**
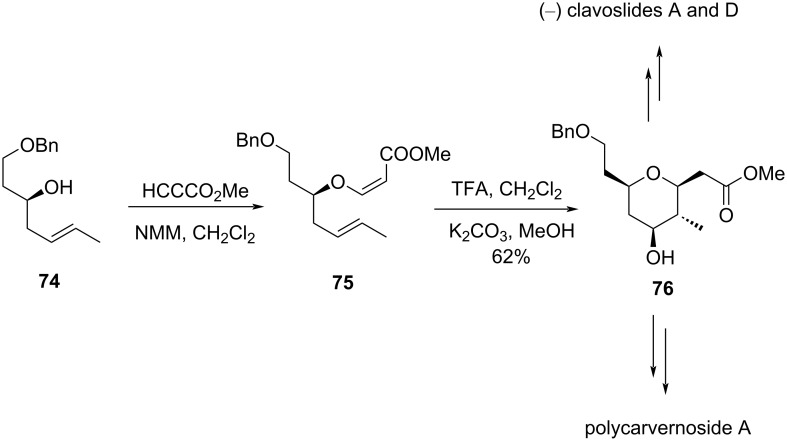
Tetrahydropyran core of polycarvernoside A as well as (−)-clavoslide A and D.

This method was utilized for the synthesis of (−)-blepharocalyxin D29 [45] and the macrolide leucascandrolide A [46]. In another type, the triflic acid-catalyzed Prins cyclization was used for the synthesis of 2,4,5,6-tetrasubstituted tetrahydropyran with complete control of stereochemistry, which is an important core of a variety of natural products, such as polycarvernoside A [47], clavoslide A [48], and (−)-blepharocalyxin [49–50] and its analogs, as shown in Scheme 16.

**Scheme 17:**
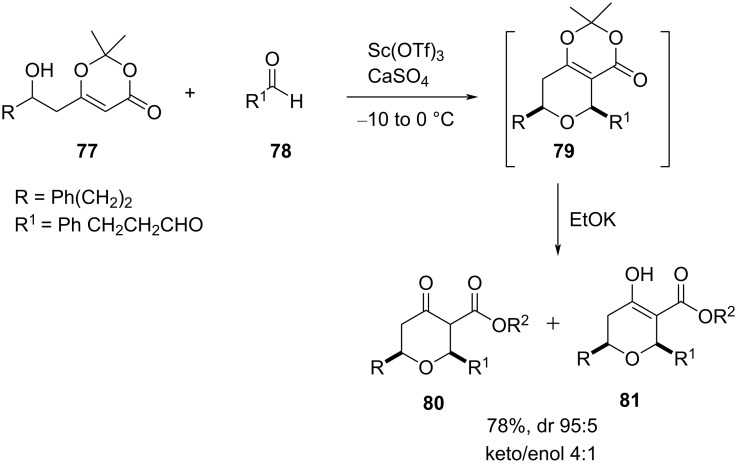
Scheidt and co-workers’ route to tetrahydropyran-4-one.

Additionally, the reaction was used for the synthesis of rhoiptelol B, 7-desmethoxyfusarentin, and corresponding analogs [51]. Considering β-hydroxydioxinone as a better starting material for Prins cyclization, Scheidt and co-workers introduced a new method to access highly functionalized chiral THP efficiently (Scheme 17) [52].

**Scheme 18:**
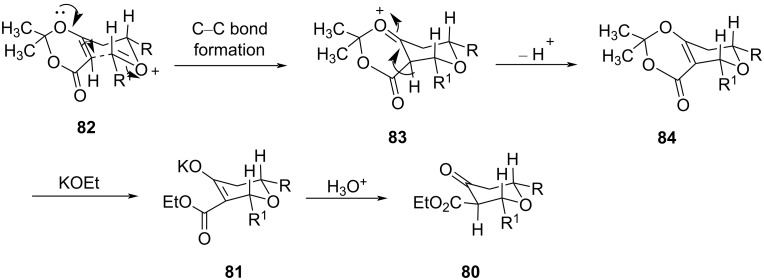
Mechanism for the Lewis acid-catalyzed synthesis of tetrahydropyran-4-one.

Furthermore, the possible reaction pathway indicates the formation of oxocarbenium ion **82**, followed by C–C bond formation via a chair-like transition state to afford **83** (Scheme 18). A sequence of reactions involving elimination of a proton from **83**, treatment of **84** with an alkoxide, and protonation of the resulting enolate delivered thermodynamically favored equatorial ester **80** and **81**.

**Scheme 19:**
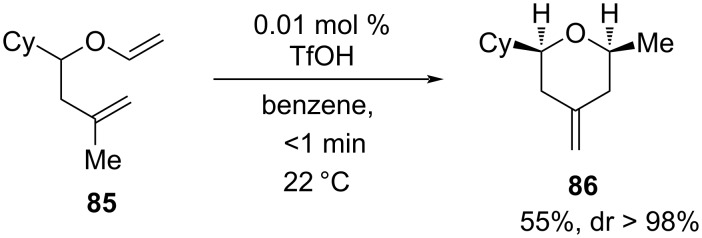
Hoveyda and co-workers’ strategy for 2,6-disubstituted 4-methylenetetrahydropyran.

The highly diastereoselective Brønsted superacid-catalyzed Prins cyclization of unsaturated enol ether **85** to *cis*-2,6-disubstituted 4-methylenetetrahydropyran **86** (55% yield) as shown in Scheme 19 was reported by Hoveyda and co-workers [53].

**Scheme 20:**
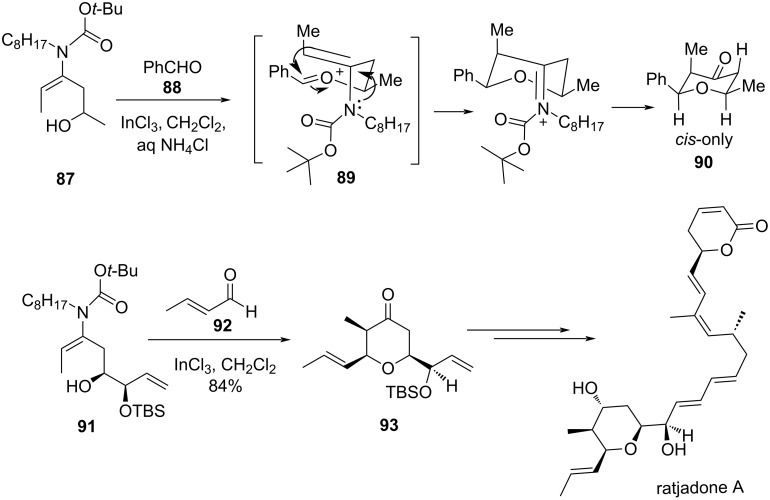
Funk and Cossey’s ene-carbamates strategy.

Funk and Cossey demonstrated that ene-carbamate could be an excellent terminating group for Prins cyclization [54]. The reaction involved **87** in the presence of the mild Lewis acid InCl_3_ and benzaldehyde (**88**), which produced all-*cis*-tetrahydropyran-4-one **90** in excellent yield. The transformation proceeded through cyclization of a diequatorial chair-like conformation of the oxocarbenium ion **89** to provide an *N*-acyliminium ion, which upon hydrolysis produced **90**. Similarly, the reaction of **91** produced all-*cis*-2,3,6-trisubstituted tetrahydropyran **93**. The application of this reaction was further extended by an exceptionally concise formal total synthesis of the nuclear export inhibitor (+)-ratjadone A, as shown in Scheme 20.

**Scheme 21:**
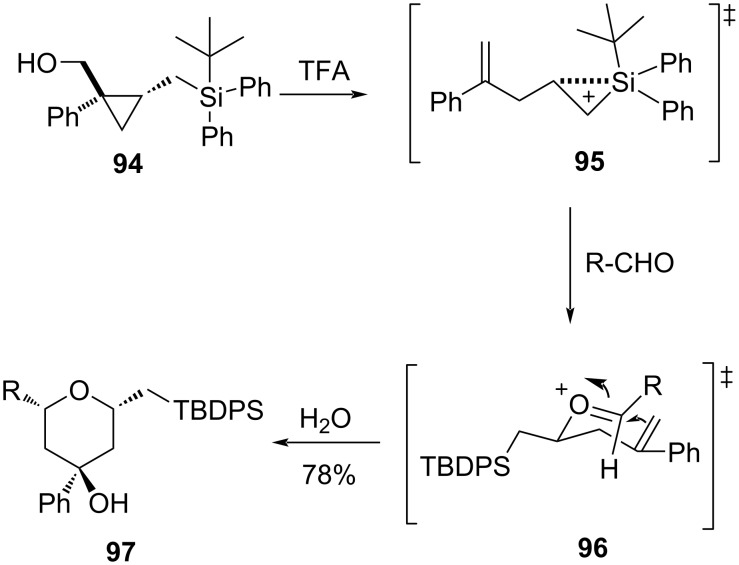
Yadav and Kumar’s cyclopropane strategy for THP synthesis.

Stereoselective Prins cyclization of substituted cyclopropylcarbinol **94** to 2,4,6-trisubstituted tetrahydropyran **97** was reported by Yadav and Kumar [55]. In this reaction, a homoallylic cation was generated from **94** by the opening of the cyclopropane ring in the presence of TFA, which upon reacting with an aldehyde delivered 2,4,6-trisubstituted tetrahydropyran **97** through Prins cyclization, as shown in Scheme 21.

**Scheme 22:**
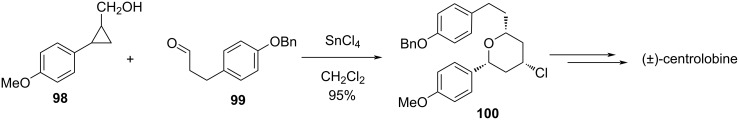
2-Arylcylopropylmethanolin in centrolobine synthesis.

Similarly, an SnCl_4_-catalyzed Prins reaction was reported for the synthesis of 4-chlorotetrahydropyran **100**. This intermediate was further utilized for the synthesis of the natural product centrolobine, as shown in Scheme 22 [56].

**Scheme 23:**
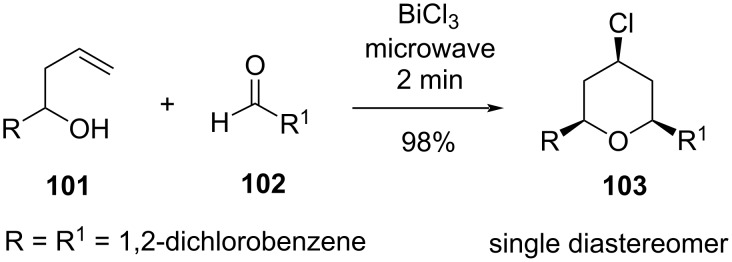
Yadav and co-workers’ strategy for the synthesis of THP.

A strategy involving BiCl_3_-catalyzed microwave-assisted Prins cyclization of homoallylic alcohol **101** with an aldehyde **102** was successfully employed for the synthesis of 4-chloro-*cis*-2,6-disubstituted tetrahydropyran **103** as a single diastereomer [57], as shown in Scheme 23.

**Scheme 24:**
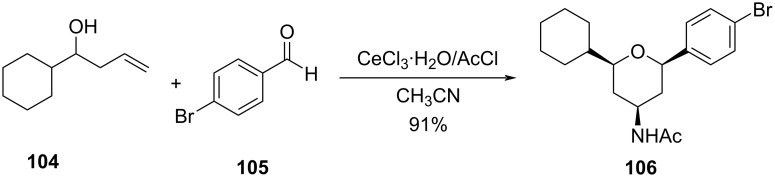
Yadav and co-workers’ Prins–Ritter reaction sequence for 4-amidotetrahydropyran.

In continuation, 4-amidotetrahydropyran derivative **106** was also synthesized from homoallylic alcohol **104** and an aldehyde **105** using a combination of cerium chloride and acetyl chloride following a Prins–Ritter reaction sequence (Scheme 24) [58]. 10 mol % cerium chloride was used as a reaction promotor, which dramatically improved the reaction rate and yield of the reaction.

**Scheme 25:**
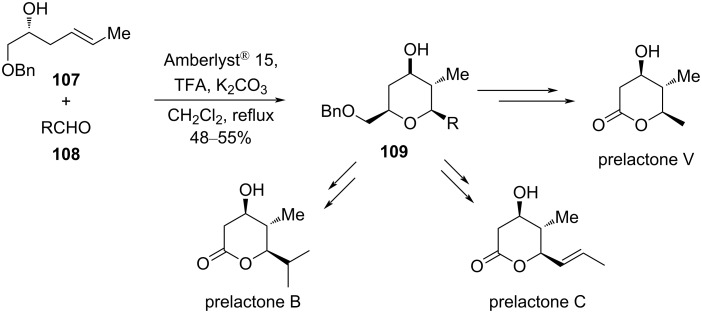
Yadav and co-workers’ strategy to prelactones B, C, and V.

In a related study, the synthesis of polysubstituted tetrahydropyrans was described by Amberlyst^®^ 15-catalyzed cyclization of homoallyl alcohol **107** and aldehydes **108**. This method was further employed for the synthesis of highly substituted tetrahydropyrans with three contiguous stereocenters in one single operation [59]. The utility of this approach is showcased in the enantioselective total synthesis of (+)-prelactones B, C, and V, as shown in Scheme 25.

**Scheme 26:**
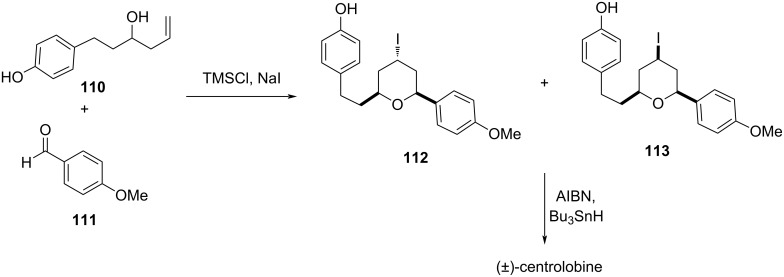
Yadav and co-workers’ strategy for the synthesis of (±)-centrolobine.

Yadav’s group reported the synthesis of 4-iodotetrahydropyrans (dr = 7.5:2.5) from aromatic aldehyde **111** and homoallylic alcohol **110** using TMSCl and NaI. Furthermore, the major diastereomer was utilized for the synthesis of centrolobine, as shown in Scheme 26 [60].

**Scheme 27:**
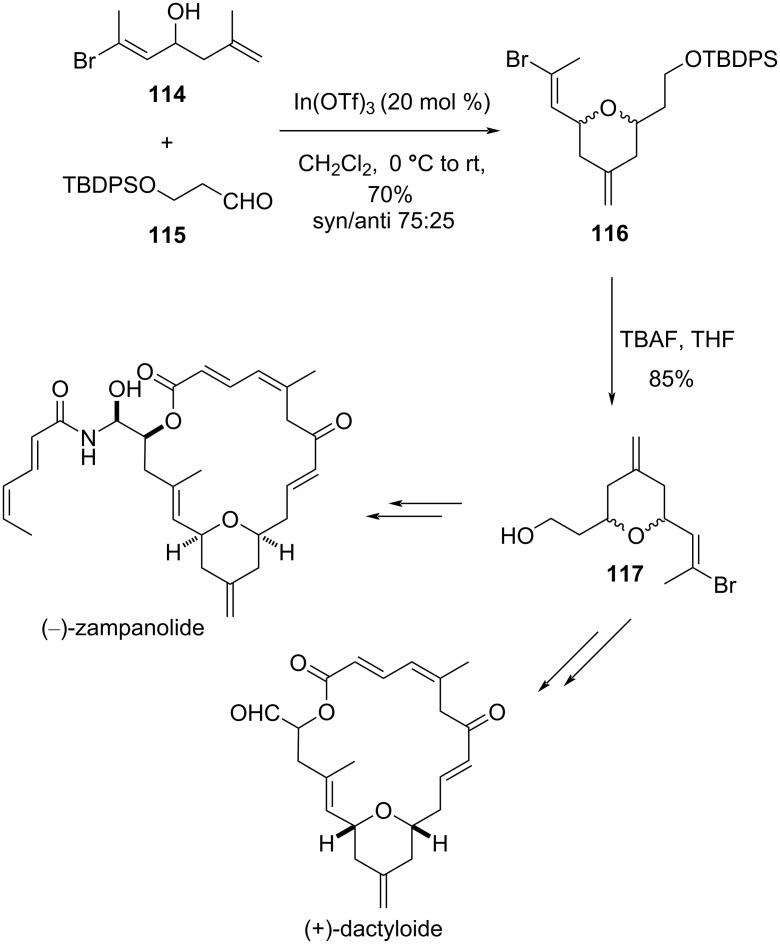
Loh and co-workers’ strategy for the synthesis of zampanolide and dactylolide.

Loh and co-workers have shown the construction of *cis*-2,6-disubstituted tetrahydropyran **116** with an exocyclic double bond by reacting homoallylic alcohol **114** and aldehyde **115** in the presence of a catalytic amount of In(OTf)_3_ [61]. This approach was further used for the synthesis of a common intermediate **117** for (−)-zampanolide and (+)-dactyloide (Scheme 27) [62].

**Scheme 28:**
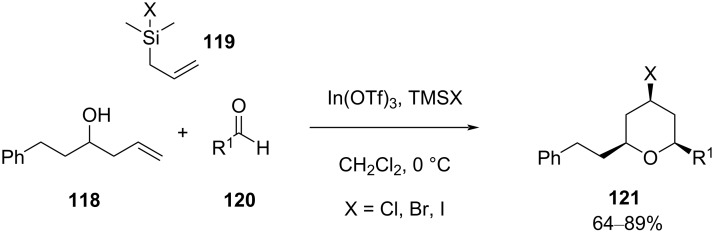
Loh and Chan’s strategy for THP synthesis.

Further improvement of this reaction was achieved by carrying out the Prins cyclization between homoallyl alcohol **118** (or using the corresponding aldehyde and allylsilyl chloride **119**) and an aldehyde **120** in the presence of a catalytic amount of the mild Lewis acid In(OTf)_3_ and trimethylsilyl halide as an additive to produce *cis*-4-halo-2,6-disubstituted tetrahydropyran **121** (Scheme 28) [63–64]. It was noticed that the problem associated with epimerization of the substrate has been successfully overcome in this reaction, which was demonstrated in the enantioselective total synthesis of (−)-centrolobine using catalytic InBr_3_ as a mild Lewis acid.

**Scheme 29:**
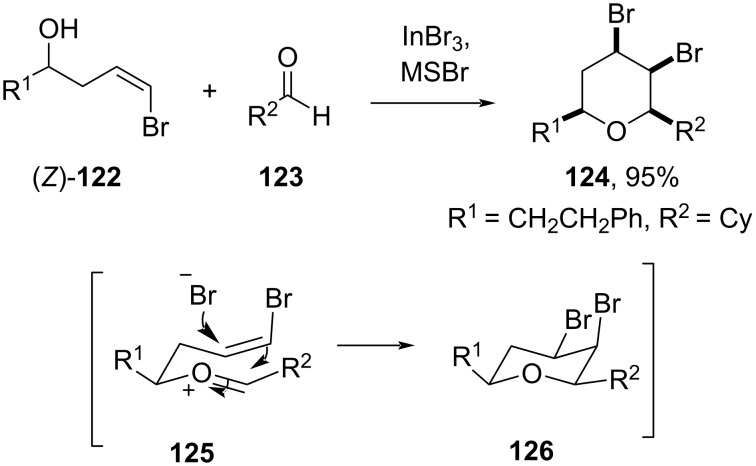
Prins cyclization of cyclohexanecarboxaldehyde.

This strategy was further explored to construct tetrasubstituted *cis*-2,6-disubstituted 4,5-dibromotetrahydropyran **124** with high stereoselectivity using γ-brominated homoallylic alcohol (*Z*)-**122** and aldehyde **123** in the presence of InBr_3_ and TMSBr in CH_2_Cl_2_ at 0 °C (Scheme 29) [65].

**Scheme 30:**
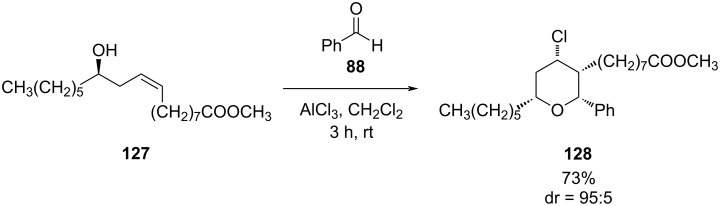
Prins cyclization of methyl ricinoleate (**127**) and benzaldehyde (**88**).

Metzger and co-workers reported an AlCl_3_-catalyzed cyclization of methyl ricinoleate (**127**) with various aldehydes to produce 2,3,6-trialkyl-substituted 4-chlorotetrahydropyran **128** with excellent stereocontrol in all-*cis*-configuration (Scheme 30) [66].

**Scheme 31:**
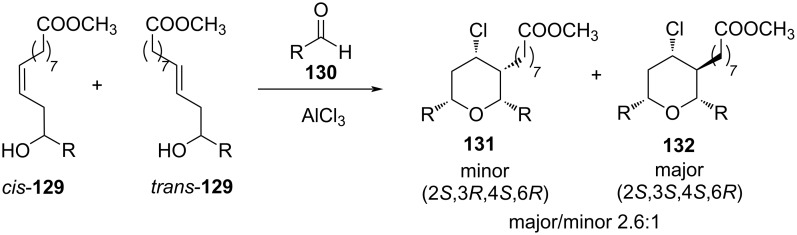
AlCl_3_-catalyzed cyclization of homoallylic alcohol **129** and aldehyde **130**.

The stereochemical outcome of this cyclization was rationalized by a chair-like transition state to produce predominantly the all-*cis* product. Similarly, *cis*- and *trans-***129** (1:4), generated in situ from methyl 10-undecenoate and an aldehyde **130** via ene reaction, undergo cyclization to form THPs **131** and **132** (Scheme 31).

**Scheme 32:**
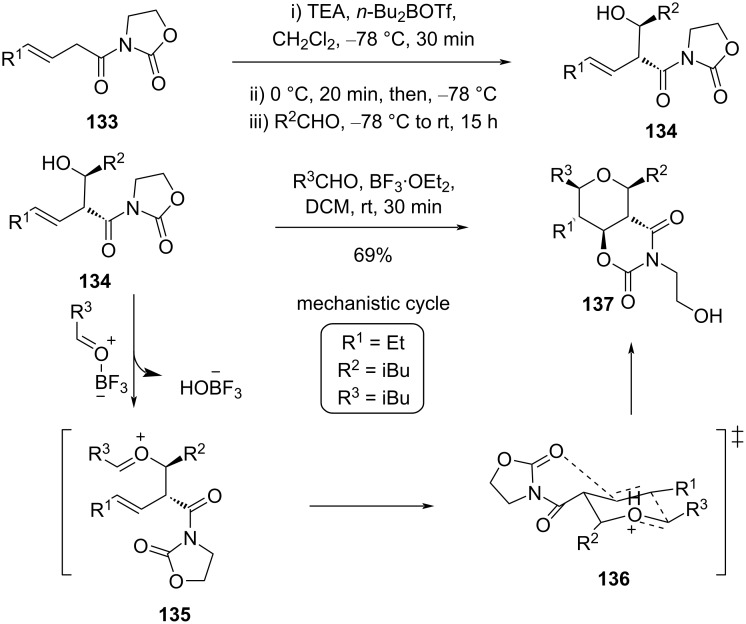
Martín and co-workers’ stereoselective approach for the synthesis of highly substituted tetrahydropyrans through an Evans aldol−Prins cyclization strategy.

Martín and co-workers reported a general strategy based on a reaction sequence of Evans aldol addition to construct a homoallylic alcohol, followed by Prins cyclization to furnish 2,3,4,5,6-pentasubstituted tetrahydropyrans **137** using β,γ-unsaturated *N*-acyloxazolidin-2-ones **134** as a key precursor [67]. In this Evans aldol−Prins (EAP) protocol, four new σ-bonds and five contiguous stereocenters were generated as shown in Scheme 32.

### Silyl-Prins cyclization

**Scheme 33:**
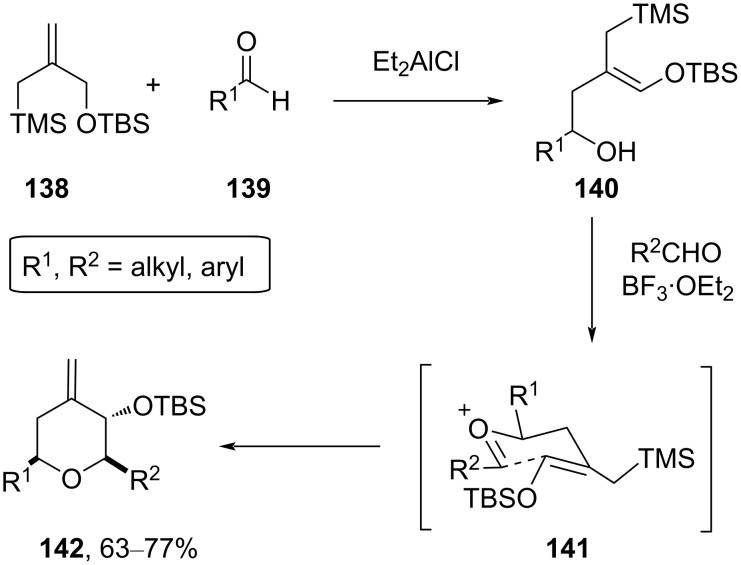
Ene-IMSC strategy by Marko and Leroy for the synthesis of tetrahydropyran.

Extensive research efforts were made towards the synthesis of THP using the silyl-Prins cyclization reaction. In this reaction, an oxocarbenium ion is being trapped by allylsilanes, vinylsilanes, alkenyl methylsilanes, or propargylsilanes to produce a variety of the Prins-cyclized products. The allyl metalation, followed by intramolecular Sakurai cyclization (IMSC) provides an efficient route to a variety of tetrahydropyran derivatives, as described by Marko and Leroy [68–69]. In these approaches, an initial ene reaction between an aldehyde **139** and the allylsilane **138** was promoted by Et_2_AlCl to generate *Z*-configured homoallylic alcohol **140**. Condensation of **140** with another aldehyde in the presence of BF_3_⋅OEt_2_ afforded the polysubstituted *exo*-methylene tetrahydropyran **142** in a completely stereocontrolled manner. The reaction proceeded via oxocarbenium **141**, which upon intramolecular trapping by the allylsilane moiety through a chair-like transition state delivers the product (Scheme 33) [68].

**Scheme 34:**
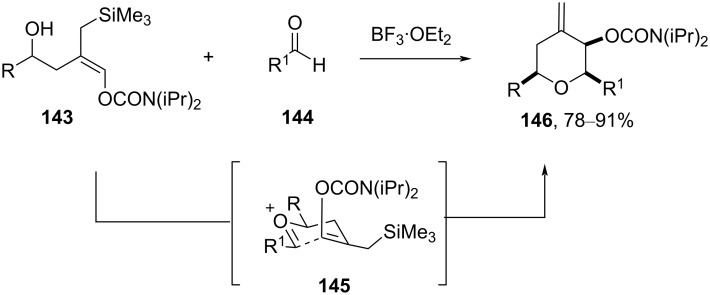
Marko and Leroy’s strategy for the synthesis of tetrahydropyrans **146**.

An analogous reaction was reported between (*E*)*-*enol carbamate **143** and an aldehyde **144** in the presence of BF_3_·OEt_2_ to provide THP **146** with exquisite diastereoselectivity. The carbamate substituent adopted the axial disposition in the proposed transition state **145**, as shown in Scheme 34 [69].

**Scheme 35:**
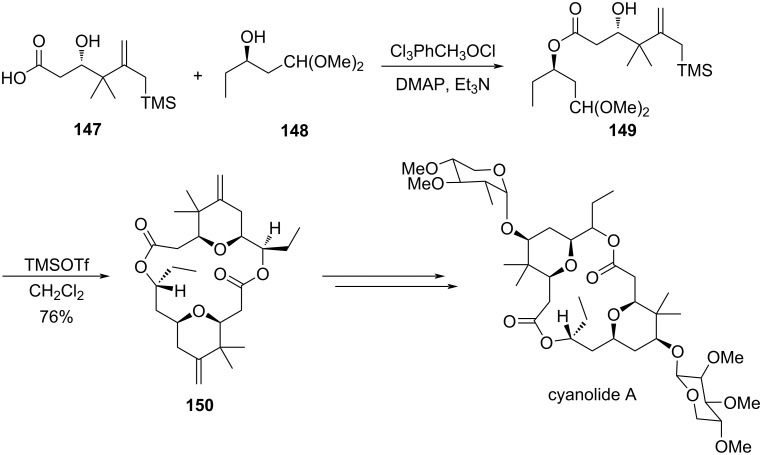
Sakurai dimerization/macrolactonization reaction for the synthesis of cyanolide A.

In another report by Rychnovsky and Gisinsky, two of the tetrahydropyran rings of the potent molluscicide cyanolide A were synthesized via a silyl-Prins cyclization and Sakurai macrocyclization/dimerization strategy to produce **150** in the presence of TMSOTf, as shown in Scheme 35 [70].

**Scheme 36:**
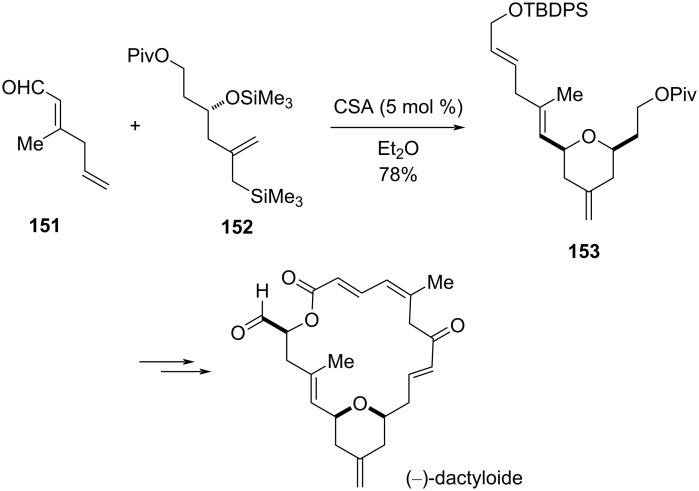
Hoye and Hu’s synthesis of (−)-dactyloide by intramolecular Sakurai cyclization.

Hoye and Hu utilized camphor sulfonic acid (CSA) to construct a *cis*-2,6-disubstituted tetrahydropyran **153** via an intramolecular Sakurai cyclization reaction between the enal **151** and an allylisilane **152**. Further manipulation of functional groups of **153** leads to the synthesis of (−)-dactyloide (Scheme 36) [71].

**Scheme 37:**
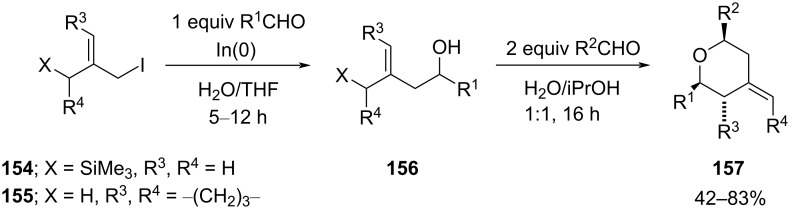
Minehan and co-workers’ strategy for the synthesis of THPs **157**.

The one-pot synthesis of a 2,6-disubstituted THP was reported by Minehan and co-workers and involved treating 3-iodo-2-[(trimethylsilyl)methyl]propene with an aldehyde in the presence of indium metal to produce homoallylic alcohol **156** (Scheme 37), which underwent a silyl-Prins cyclization to form polysubstituted *exo*-methylene THPs **157** [72].

### Tandem allylation–silyl-Prins cyclization

**Scheme 38:**
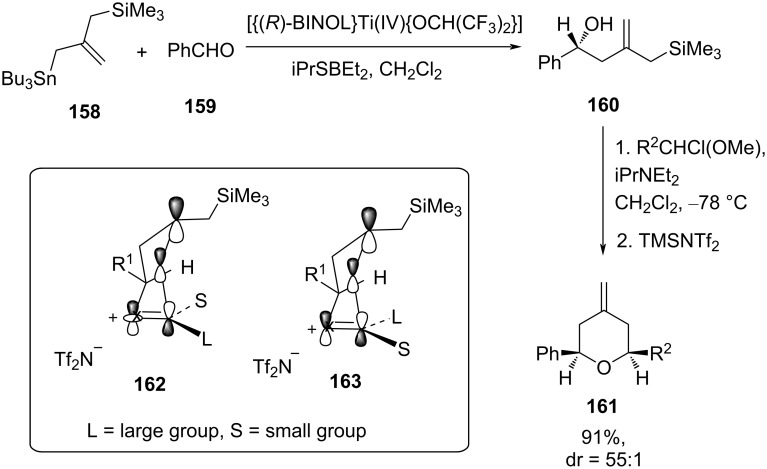
Yu and co-workers’ allylic transfer strategy for the construction of tetrahydropyran **161**.

Tetrahydropyran can also be synthesized stereoselectively by sequential allylation to an aldehyde, followed by silyl-Prins cyclization of the resulting homoallylic alcohol. For illustration, a facile enantioselective strategy for the synthesis of *cis*-2,6-disubstituted 4-methylenetetrahydropyran **161** (91% yield, dr = 5:1) was reported by Yu et al, utilizing, first, asymmetric allylation of an aldehyde by using [{(*R*)-BINOL}Ti(IV){OCH(CF_3_)_2_}_2_] as a chiral promotor in PhCF_3_, followed by cyclization using R_2_CHCl(OMe) in the presence of TMSNTf_2_, as shown in Scheme 38 [73]. The internal chirality transfer during cyclization probably took place due to the geometrical preference of **162** to minimize the allylic strain with the existing stereogenic center (pseudoaxial group), leading to the formation of *cis*-tetrahydropyran **161** rather than a *trans*-tetrahydropyran.

**Scheme 39:**
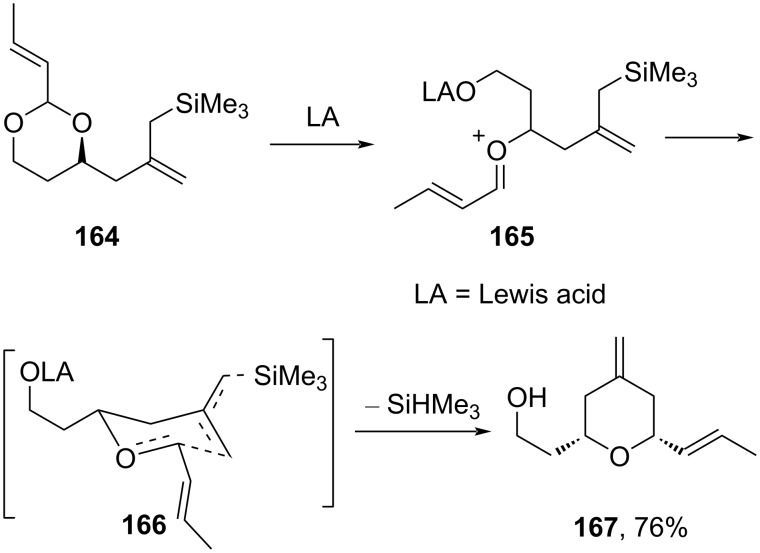
Reactivity enhancement in intramolecular Prins cyclization.

Floreancig and co-workers utilized a tandem allylation–silyl-Prins cyclization strategy to afford 2,6-disubstituted tetrahydropyran **167** by ionizing α,β-unsaturated acetals **164** in the presence of electron-rich olefins using Ce(NO_3_)_3_ and SDS in water [74]. The mechanism of the reaction is shown in Scheme 39, which plausibly proceeded through trapping of oxocarbenium ion **166** in a chair-like transition state.

**Scheme 40:**
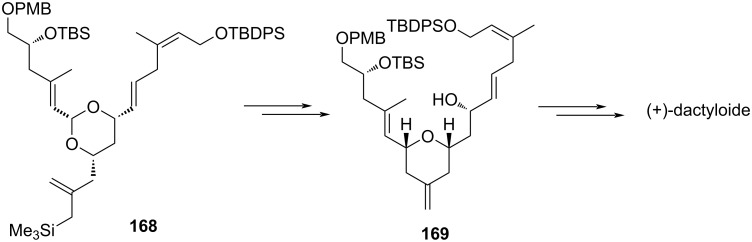
Floreancig and co-workers’ Prins cyclization strategy to (+)-dactyloide.

The stability of the acetal under these reaction conditions reflected that the acid-sensitive functional groups are well tolerated in the cyclized product. Furthermore, a natural product, (+)-dactyloide, was synthesized by following the above strategy using an appropriate acetal (Scheme 40) [75].

**Scheme 41:**
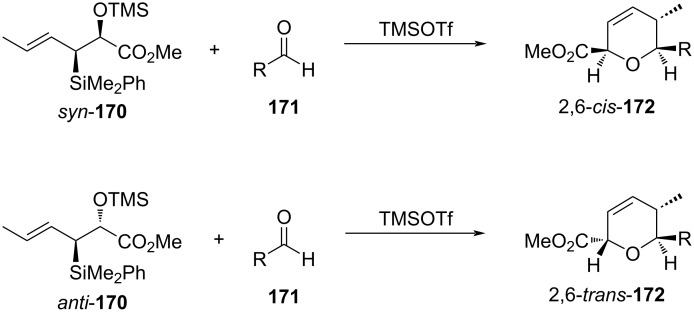
Panek and Huang’s DHP synthesis from crotylsilanes: a general strategy.

The synthesis of enantiomerically enriched **172**, *cis*-2,6-DHP and *trans*-2,6-DHP, respectively, was reported by a [4 + 2]-annulation strategy. The authors utilized crotylsilanes *syn*-**170** and *anti*-**170**, respectively, with an aldehyde **171** in the presence of TMSOTf to deliver different DHPs **172** (Scheme 41) [76].

**Scheme 42:**
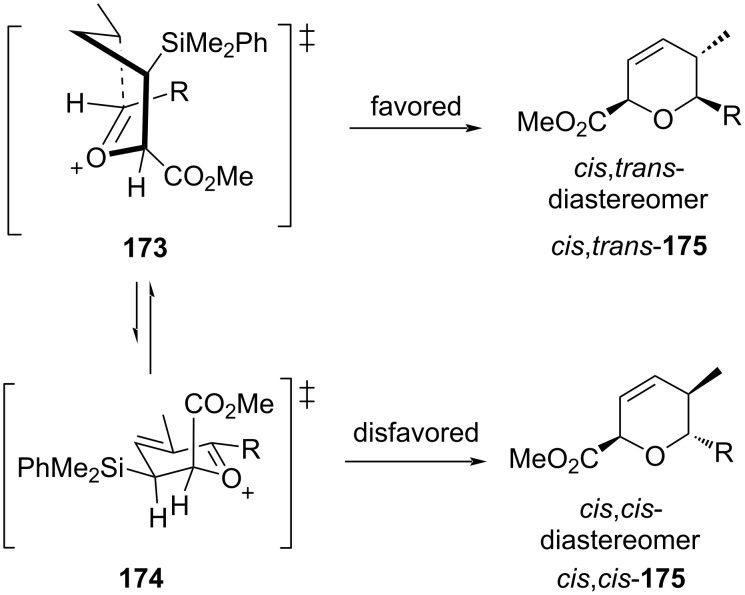
Panek and Huang’s DHP synthesis from *syn*-crotylsilanes.

For *syn*-**170**, the reaction went via the favored boat-like transition state **173** instead of the disfavored chair-like transition state **174** to *cis*,*trans*-**175** as a product (Scheme 42).

**Scheme 43:**
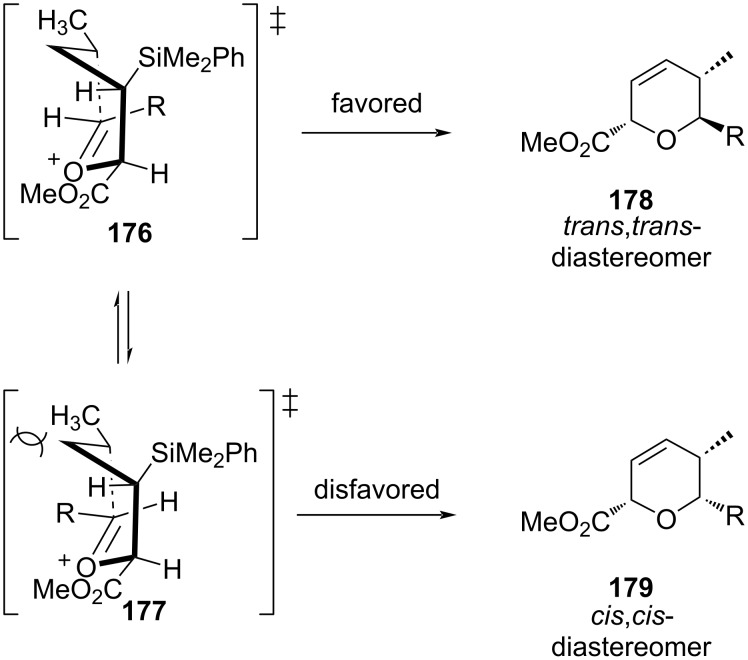
Panek and Huang’s DHP synthesis from *anti*-crotylsilanes.

In contrast, the reaction of *anti*-**170** proceeded, however, through similar boat-like transition states **176** and **177** where the interaction between the methyl substituent and the alkyl group of aldehyde was less, leading to the formation of *trans*,*trans*-**178** as a major product, as shown below in Scheme 43.

**Scheme 44:**
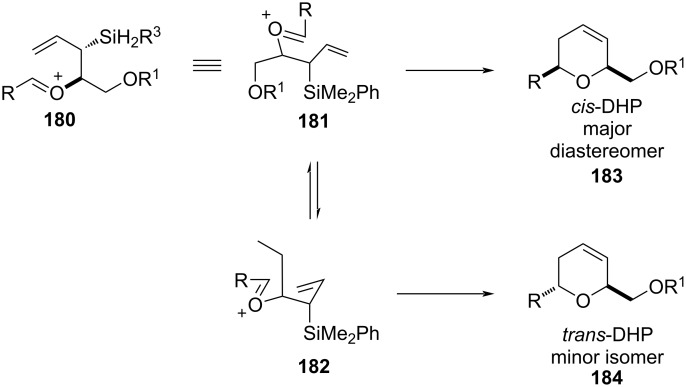
Roush and co-workers’ [4 + 2]-annulation strategy for DHP synthesis [82].

A variety of natural products, such as (−)-apicularen A [77], the C1–C13 fragment of bistramide A71 [78], herboxideiene/GEX1A [79], (+)-kendomycin [80], and (+)-SCH351448 [81] were synthesized utilizing this [4 + 2]-annulation strategy. Following the above annulation route, later, Roush's group Introduced β-hydroxyallylsilanes for the synthesis of 2,6-disubstituted DHP (Scheme 44) [82].

**Scheme 45:**
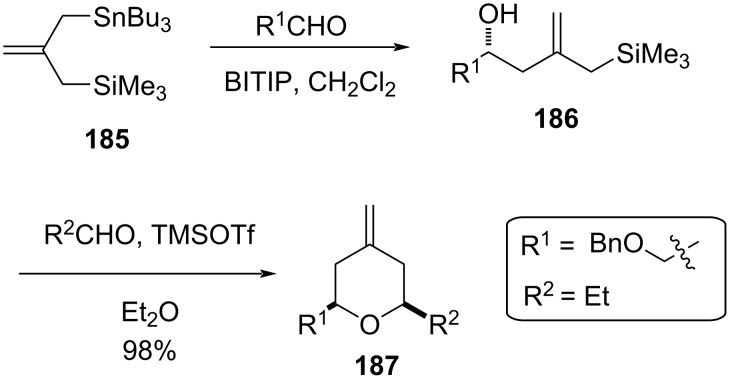
TMSOTf-promoted annulation reaction.

This strategy was further utilized for the synthesis of the C29−C45 bispyran subunit (E−F) of spongistatin [82]. 2,6-Disubstituted 4-methylenetetrahydropyran was also synthesized from silylstannane and two units of aldehyde in a two-step protocol. The first step involves the addition between silylstannane **185** and an aldehyde in the presence of titanium BINOLate as a catalyst, which afforded hydroxyallylsilane **186** with excellent enantioselectivity (Scheme 45) [83–85]. This, upon further reaction with another aldehyde in the presence of TMSOTf, gave 2,6-disubstituted 4-methylenetetrahydropyran **187**. This strategy was utilized for the synthesis of bryostatin and (+)-dactyloide analogs [86–88].

**Scheme 46:**
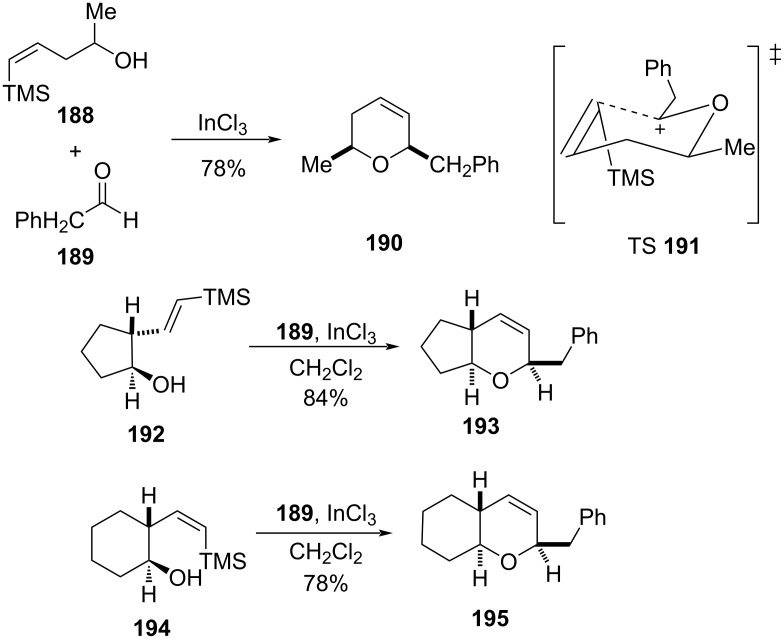
Dobb and co-workers’ synthesis of DHP.

Similar to Prins cyclization of allylsilanes, Dobbs and co-workers recently utilized the corresponding vinylsilane as an alternative for the synthesis of *cis*-2,6-dihydropyran [89–90]. The synthesis involves tandem addition of vinylsilane, followed by silyl-Prins cyclization reaction. For example, 4-trimethylsilylpent-4-en-2-ol (**188**), upon reaction with phenylacetaldehyde (**189**) in the presence of InCl_3_, gave *cis*-2,6-dihydropyran **190** via chair-like transition state **191**. This strategy was further elaborated for the synthesis of 5,6- and 6,6-ring-fused dihydropyrans **193** and **195**, respectively, as shown in Scheme 46.

**Scheme 47:**
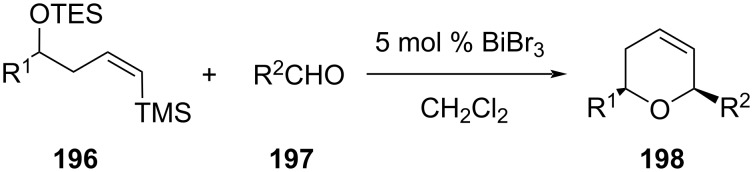
BiBr_3_-promoted tandem silyl-Prins reaction by Hinkle et al.

A similar tandem strategy of an addition of vinylsilane **196**, followed by silyl-Prins cyclization with an aldehyde **197** in the presence of 5 mol % BiBr_3_, was reported by Hinkle and co-workers to give the corresponding compound **198** (Scheme 47) [18].

**Scheme 48:**
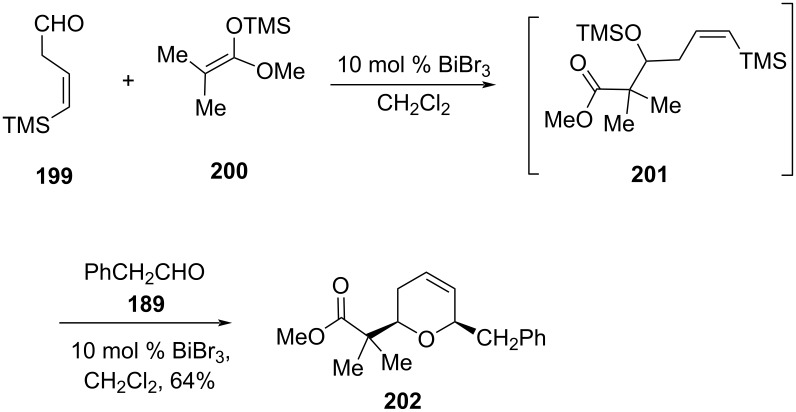
Substrate scope of Hinkle and co-workers’ strategy.

The authors further investigated the Mukaiyama aldol reaction between the β,γ-unsaturated aldehyde **199** and acetal **200** in the presence of 10 mol % BiBr_3_ to obtain aldol product **201**. However, the addition of 2 equiv of phenylacetaldehyde (**189**) and 10 mol % BiBr_3_ afforded dihydropyran **202** in 64% yield as a single isomer, as shown in Scheme 48 [91].

**Scheme 49:**
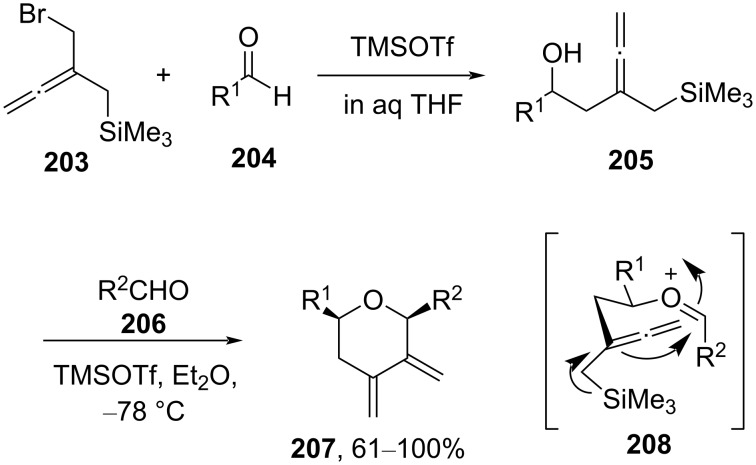
Cho and co-workers’ strategy for 2,6 disubstituted 3,4-dimethylene-THP.

The *cis*-2,6-disubstituted tetrahydropyran **207** with two adjacent methylene groups at the C3 and C4 positions was synthesized via silyl-Prins cyclization of silane **205** with an aldehyde in the presence of Lewis acid TMSOTf [92]. The reaction proceeded through transition state **208** following silyl-Prins cyclization, as shown in Scheme 49.

**Scheme 50:**
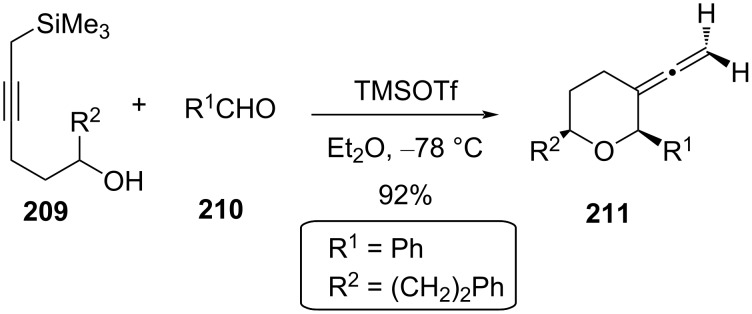
Furman and co-workers’ THP synthesis from propargylsilane.

Unlike allyl- and vinylsilanes, as discussed earlier, Furman and co-workers introduced a new concept of synthesizing **211** utilizing silyl-Prins cyclization of propargylsilane **209** and aldehyde **210** in the presence of TMSOTf [93]. The oxocarbenium ion was intramolecularly trapped by the olefin, followed by removal of trimethylsilane (Scheme 50).

**Scheme 51:**
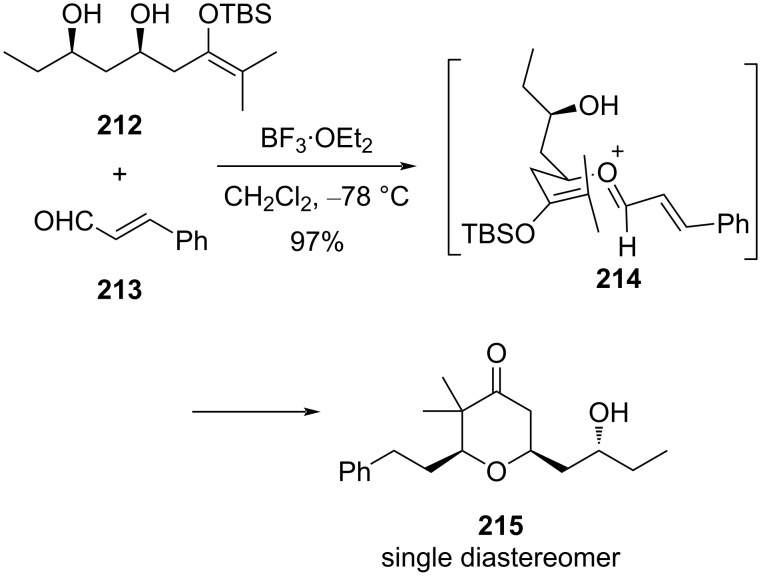
THP synthesis from silyl enol ethers.

The authors further explored this strategy for the asymmetric synthesis of 3-vinylidene-substituted tetrahydropyran by taking the chiral propargylsilane. A diastereoselective route to *cis*-2,6-disubstituted tetrahydropyran-4-one **215** was explored by introducing a silyl enol ether Prins cyclization concept in which oxocarbenium ion **214**, generated by reacting hydroxy-substituted silyl enol ether **212** with aldehyde **213** (different types of aliphatic and aromatic as well as α,β-unsaturated aldehydes were used), was trapped by silyl enol ether [94]. A detailed mechanism similar to simple Prins cyclization, except trapping of oxocarbenium ion **214** with silyl enol ether instead of olefin, vinylsilane, or allylsilanes, was proposed as shown in Scheme 51.

**Scheme 52:**
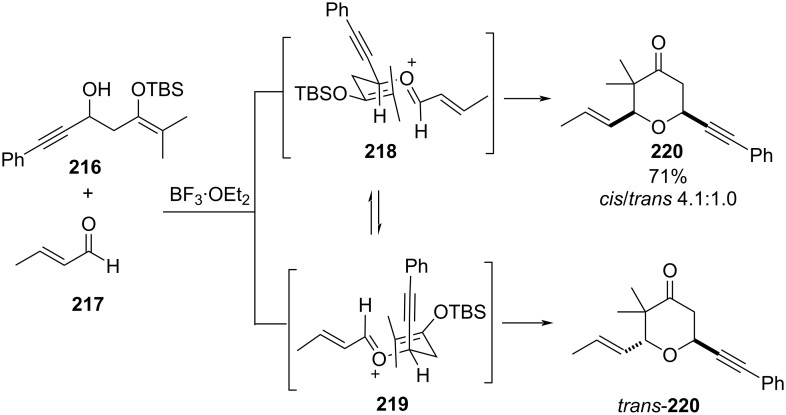
Rychnovsky and co-workers’ strategy for THP synthesis from hydroxy-substituted silyl enol ethers.

However, the reaction of silyl enol ether such as **216**, upon reacting with an unsaturated aldehyde **217**, produced a mixture of *cis*- and *trans*-**220** (dr = 4.1:1.0). It was explained that the diastereoselectivity of the product depends on the size of the substituent. For example, when the substituent is sterically small, it occupies the pseudoaxial position in the reactive conformation **218** (Scheme 52).

**Scheme 53:**
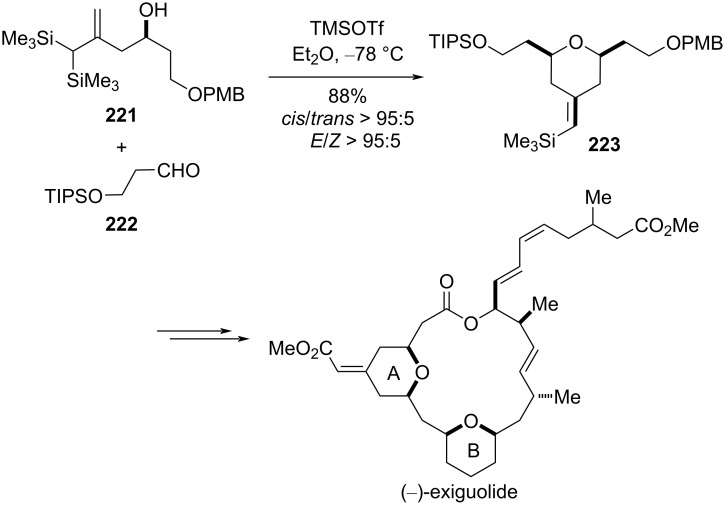
Li and co-workers’ germinal bissilyl Prins cyclization strategy to (−)-exiguolide.

Li et al. utilized allylic geminal bissilyl alcohol **221** for the construction of THP ring A of (−)-exiguolide via Prins cyclization with an aldehyde in the presence of Lewis acid as a promoter [95]. High yield and excellent diastereoselectivity were obtained under standard silyl-Prins cyclization conditions using TMSOTf as Lewis acid (Scheme 53).

**Scheme 54:**
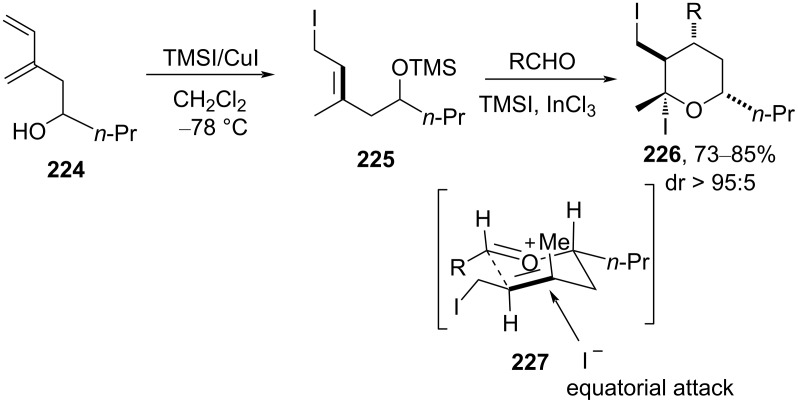
Xu and co-workers’ hydroiodination strategy for THP.

Recently Xu et al. reported the homoallylic silyl alcohol **224** containing a multisubstituted (*Z*)-alkene reacting with an aldehyde in the presence of TMSI and InCl_3_ to afford **226** in high diastereoselectivity [96]. The authors assumed that the Prins cyclization proceeded through Alder’s chair-like transition state **227** in which the (*Z*)-alkene accounts for the *trans*-stereocontrol at the C3 position and equatorial iodide addition accounts for the *cis*-stereocontrol at the C4 position, as shown below in Scheme 54.

**Scheme 55:**
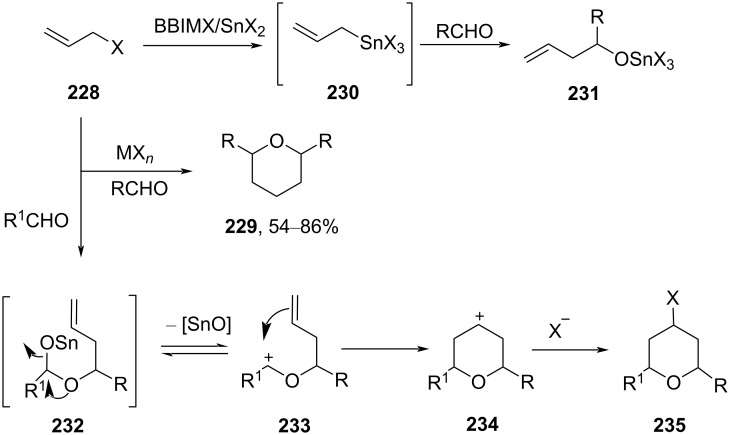
Wang and co-workers’ strategy for tetrahydropyran synthesis.

The one-pot synthesis of tetrahydropyran by utilizing the Babier–Prins cyclization reaction of allyl bromide (**228**) with a carbonyl compound promoted by BBIMBr/SnBr_2_ complex under solvent-free conditions has been explored [97]. The mechanism of the reaction was shown to include a Barbier reaction of allyl bromide with an aldehyde in the presence of SnBr_3_ and a quaternary ammonium salt to produce allyltin compound **230**, which subsequently reacts with an aldehyde to generate intermediate **231**. This intermediate could be hydrolyzed by water during workup to afford **232**, which does not give the required THP product. Desired product **235** was obtained only in the anhydrous conditions (Scheme 55).

**Scheme 56:**
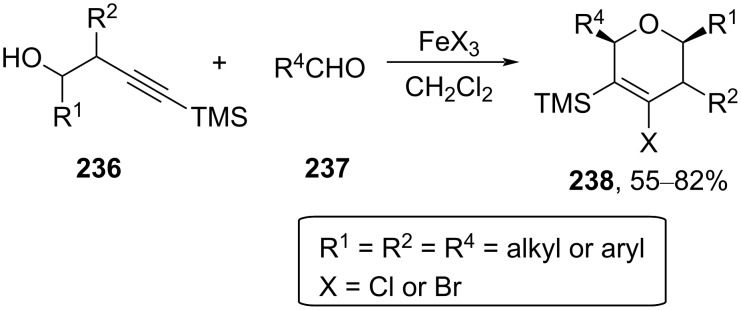
FeCl_3_-catalyzed synthesis of DHP from alkynylsilane alcohol.

The methodology of alkynylsilane Prins cyclization was explored for the synthesis of 2,6-dihydropyran **238** by reacting secondary homopropargyl alcohol **236**, having a trimethylsilyl group at the triple bond, with an aldehyde (Scheme 56) [98–101]. The reaction follows alkyne Prins cyclization and minimizes the competitive 2-oxonia-[3,3]-sigmatropic rearrangement pathway. The reaction was highly stereoselective and afforded the *cis*-2,6-dihydropyran in the presence of Lewis acid FeCl_3_.

**Scheme 57:**
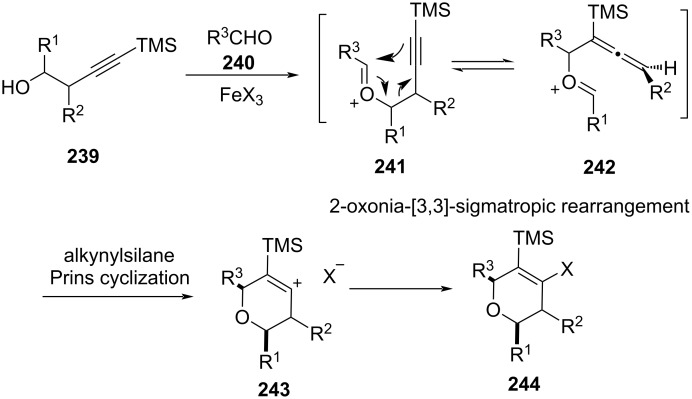
Martín, Padrón, and co-workers’ proposed mechanism of alkynylsilane Prins cyclization for the synthesis of DHP.

From DFT calculations, the authors concluded that the Prins product is formed more rapidly than the α-trimethylsilylalkenyl cation **242** formed by the Grob-type fragmentation (Scheme 57), which was trapped by the subsequent attack of the halide anion, leading to the formation of Prins product **244**. On the basis of theoretical calculations, the authors could conclude factors controlling the alkyne Prins cyclization over formal 2-oxonia-[3,3]-sigmatropic rearrangement.

**Scheme 58:**
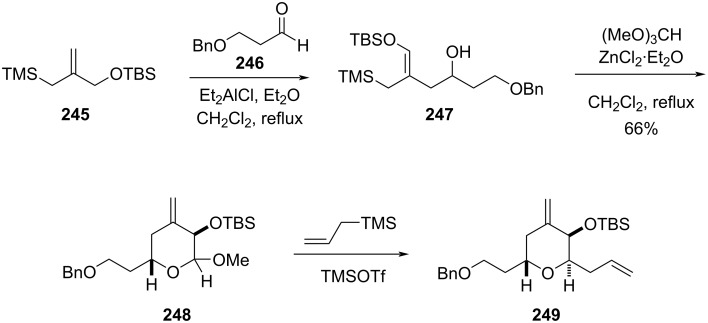
Marko and co-workers’ synthesis of 2,6-*anti*-configured tetrahydropyran.

Furthermore, Markó and co-workers successfully synthesized 2,6-*anti*-configured THP starting from allylsilane **245**, following diethylaluminium chloride-promoted ene reaction and condensation with an aldehyde **246** [102]. Expected ene adduct **247** was obtained as a (*Z*)-olefin. The addition of ZnCl_2_·Et_2_O and (MeO)_3_CH to the resulting homoallylic alcohol **247** leads to the desired pyran derivative **248**, having an acetal group at the C2 position (Scheme 58). By treatment of acetal **248** with allyltrimethylsilane gave 2,6-*anti*-configured THP **249** as a single diastereomer in the presence of TMSOTf.

**Scheme 59:**
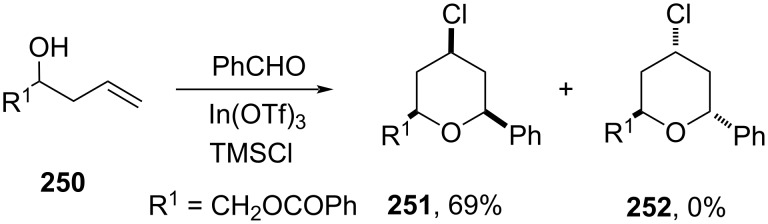
Loh and co-workers’ strategy for 2,6-*syn*-tetrahydropyrans.

A new route to obtain 2,6-*anti*-configured THP ring **252** was reported using homoallylic α-hydroxy ester **250** in an In(OTf)_3_-catalyzed Prins cyclization with moderate selectivity. Although selectivity was not observed (almost 1:1), a variety of 2,6-*syn* and *anti*-4-chloro-trisubstituted THPs were prepared depending upon nature of R^1^ in **250**. Whereas, particularly with benzoyl ester substituent (**250**), only *syn* product **251** was obtained in 69% yield (Scheme 59) [103].

**Scheme 60:**
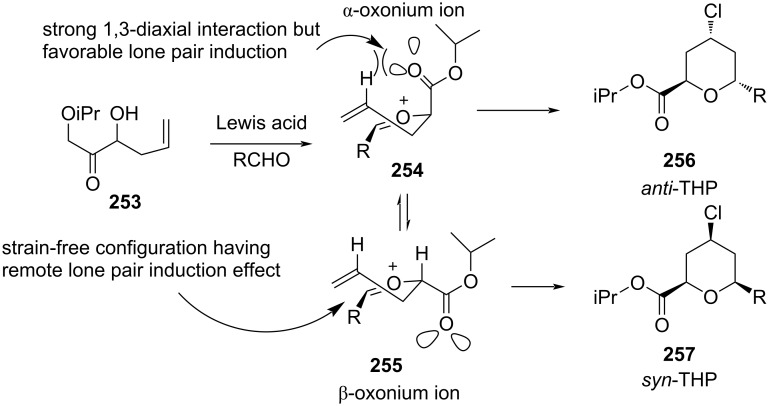
Loh and co-workers’ strategy for *anti*-THP synthesis.

The possible mechanism for the formation of a variety of isomers was explained through transition state **254** and **255** (Scheme 60). Competition between electronically favored transition state **254** leads to the formation of *anti*-isomer **256**, whereas the sterically preferred transition state **255** afforded *syn*-isomer **257**.

**Scheme 61:**
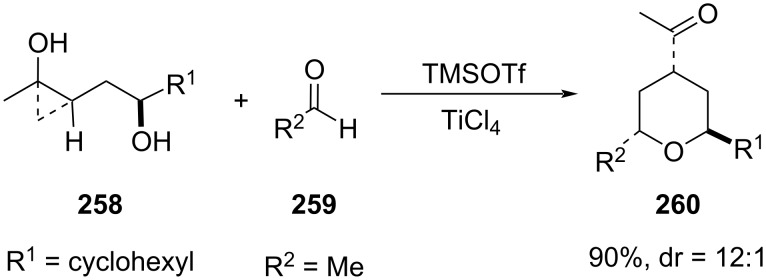
Cha and co-workers’ strategy for *trans*-2,6-tetrahydropyran.

Unlike the well-explored selective synthesis of major *cis*-2,6-THP, a highly stereoselective route to the thermodynamically disfavored *trans*-2,6-tetrahydropyran **260** was reported by Cha and co-workers based on the coupling of hydroxyethyl-tethered cyclopropanol **258** and aliphatic aldehyde **259** using TiCl_4_ as a Lewis acid [104–105]. The reaction proceeded through the Prins cyclization (Scheme 61).

**Scheme 62:**
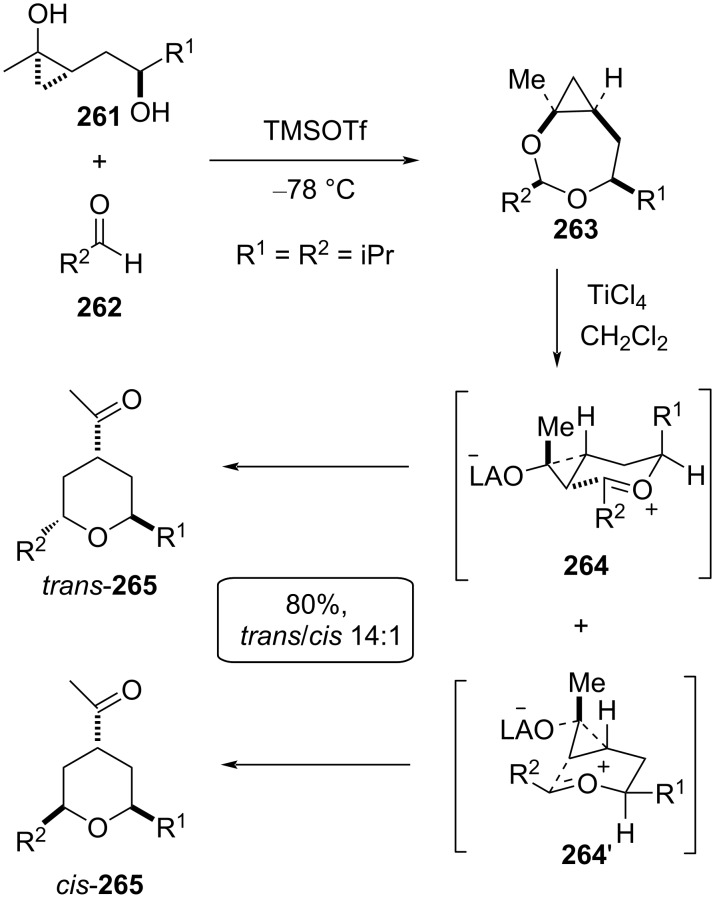
Mechanism proposed by Cha et al.

The reaction proceeded via formation of a 7-membered cyclic acetal **263** as a single isomer in nearly quantitative yield, followed by Lewis acid-catalyzed rearrangement leading to the formation of tetrahydropyran. Under optimized reaction conditions, TMSOTf gave 7-membered cyclic acetal **263**, which upon treatment with TiCl_4_ gave the desired THP as a 14:1 mixture of *trans*- and *cis*-**265** in 80% yield. The *trans*-**265** was obtained as a major isomer, where the reaction proceeded through the 6-membered chair-like transition state **264**, and the electrophilic ring opening of cyclopropane by the oxocarbenium ion was believed to proceed via “corner attack” at the less substituted C–C bond. However, minor *cis*-**265** was formed via the 6-membered boat like transition state **264’** (Scheme 62) [104].

**Scheme 63:**
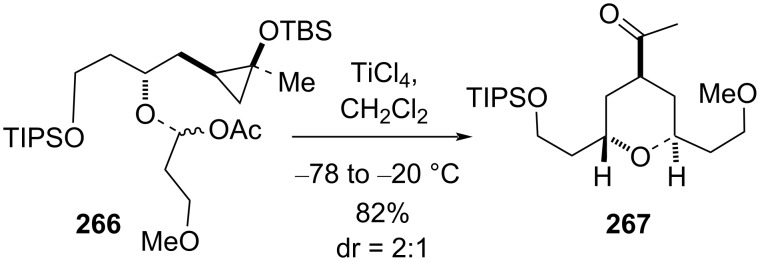
TiCl_4_-mediated cyclization to *trans*-THP.

Cha’s group also utilized the Rechnovsky convergent method where an α-acetoxy ether was used as a precursor for the oxocarbenium ion in the THP synthesis to complement the aforementioned 7-membered cyclic acetal strategy. The treatment of α-acetoxy ether **266** with Lewis acid produced the corresponding THP **267** with moderate diastereoselectivity in favor of the *trans*-2,6-stereoisomer, as shown in Scheme 63.

**Scheme 64:**
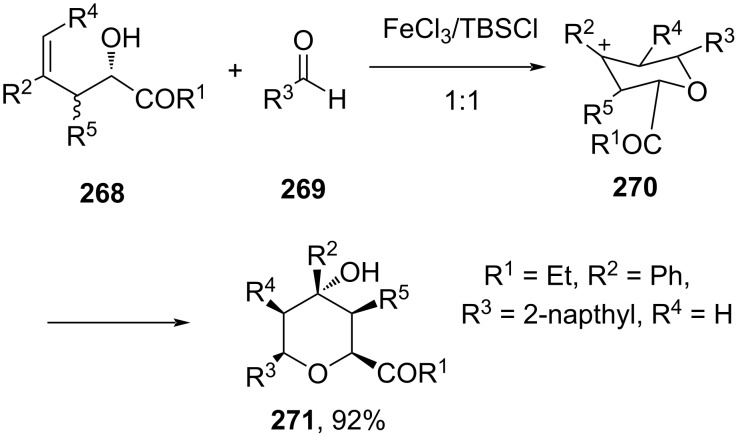
Feng and co-workers’ FeCl_3_-catalyzed Prins cyclization strategy to 4-hydroxy-substituted THP.

A variety of 4-hydroxy-substituted THPs was exclusively generated via Prins reaction using FeCl_3_ as a Lewis acid catalyst. Excellent stereoselectivity was obtained for a remarkably broad range of substrates under mild reaction conditions (Scheme 64) [106].

The authors proposed fundamental insights into the mechanism of the reaction based on DFT calculations. A different [2 + 2]-cycloaddition process was suggested to rationalize the observed OH-selectivity.

**Scheme 65:**
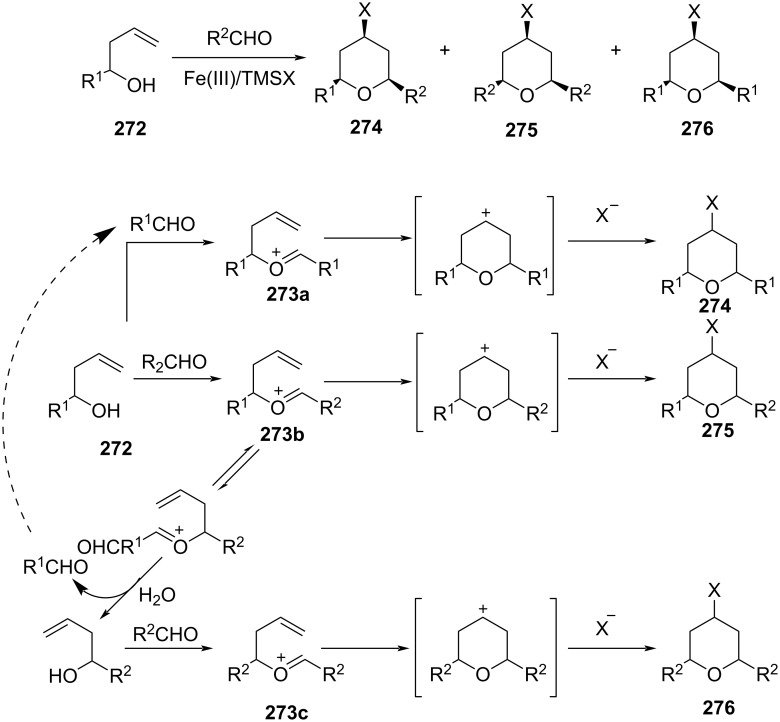
Selectivity profile of the Prins cyclization under participation of an iron ligand.

In 2015, Padrón and co-workers also reported the Prins cyclization catalyzed by a Fe(III) and trimethylsilyl halide system for the synthesis of all-*cis*-2,4,6-trisubstituted THP [107]. As reported previously by Feng et al. [106], two mechanistic pathways via the classical oxocarbenium route and [2 + 2]-cycloaddition were considered for DFT calculations. Experimental and DFT studies suggested the preference of a classical oxocarbenium route over the [2 + 2]-pathway for those alcohols having unactivated and unsubstituted alkenes, whereas the substituent adjacent to the hydroxy group in the homoallylic alcohol controls the oxonia-Cope rearrangement (see **273a**–**c**). The alkyl substituent favored the exclusive formation of crossed THP derivatives, whereas 2-oxonia-Cope rearrangement was thermodynamically favored in the presence of a phenyl group (Scheme 65).

**Scheme 66:**
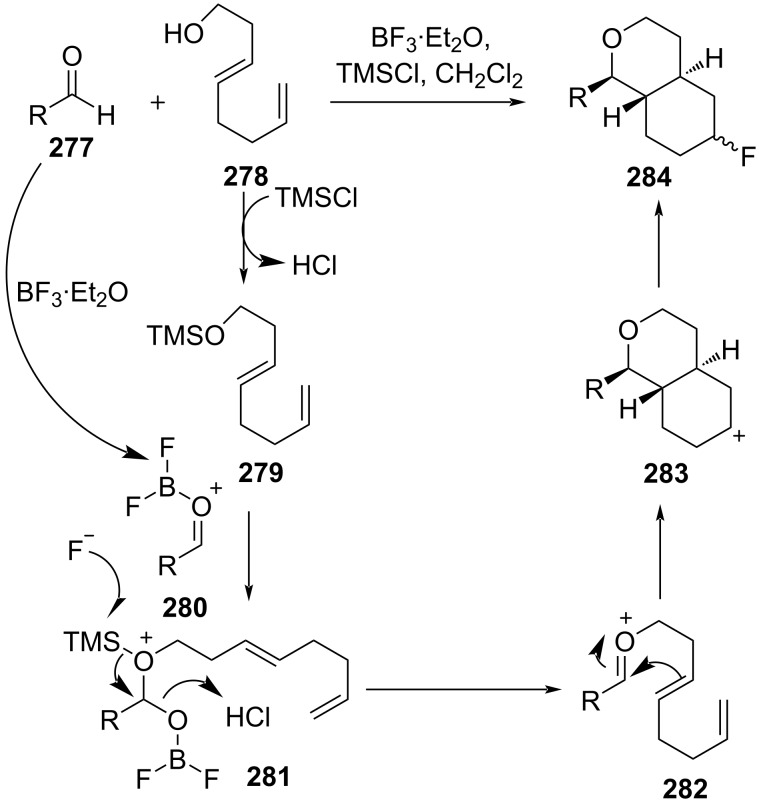
Sequential reactions involving Prins cyclization.

Matsumoto and co-workers reported a Lewis acid-mediated Prins cyclization between an alcohol **278** bearing a nonconjugated diene moiety and an aldehyde **277** with alkyl or aryl substituent in presence of BF_3_·Et_2_O and TMSCl at −40 °C to afford corresponding fluorinated bicyclic compound **284** [108]. A catalytic amount of TMSCl generates TMS-protected alcohol **279** and HCl. The activated aldehyde **280** reacts with **279** to form the intermediate **281**. Then, the TMS group in **281** is attacked by F^−^ in the presence of HCl to give the alkoxycarbenium ion intermediate **282**, which is followed by a sequential cyclization to form secondary carbocation **283**, which in the presence of fluoride ions affords **284**, as shown in Scheme 66.

**Scheme 67:**
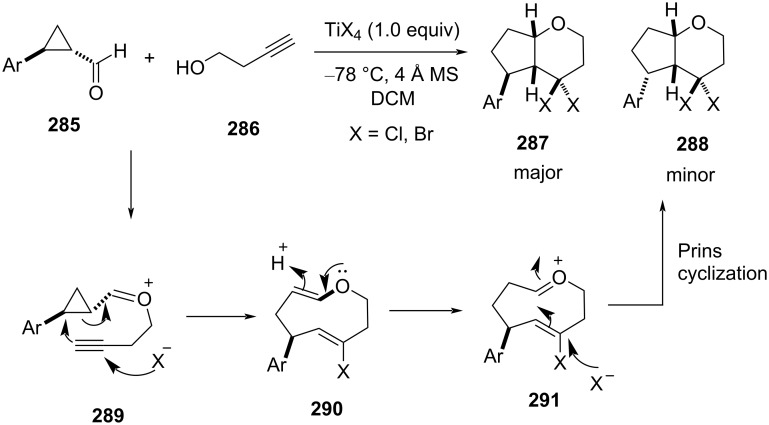
Banerjee and co-workers’ strategy of Prins cyclization from cyclopropane carbaldehydes and propargyl alcohol.

Banerjee et al. explored the reactivity of cyclopropane carbaldehydes **285** with 3-butyn-1-ol in the presence of TiX_4_ for the stereoselective construction of the THF ring (Scheme 67) [109]. A series of geminal bishalogen-containing fused THPs was synthesized in high yield (up to 80%) and excellent diastereoselectivity. A Prins cyclization mechanism was proposed for the above transformation in the presence of TiCl_4_. Formation of the oxocarbenium ion **289**, followed by an intramolecular nucleophilic attack by the alkynyl bond on the cyclopropane unit gave cyclic oxocarbenium intermediate **290**. Further, the attack of a halide anion (from TiX_4_) leads to the Prins cyclization to give bishalogenated bicyclic THP with all-*cis*-stereochemistry in the major product.

### Asymmetric Prins cyclization

**Scheme 68:**
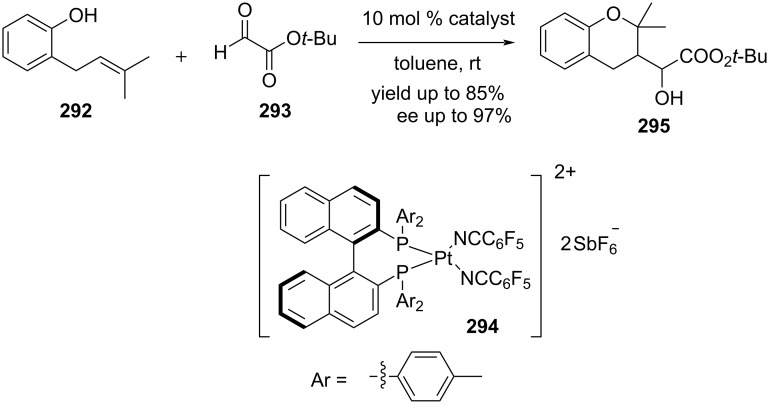
Mullen and Gagné's (*R*)-[(tolBINAP)Pt(NC_6_F_5_)_2_][SbF_6_]_2_-catalyzed asymmetric Prins cyclization strategy to chromans.

Mullen and Gagné reported a first catalytic asymmetric Prins cyclization reaction between 2-allylphenol **292** and glyoxylate ester **293** using (*R*)-[(tolBINAP)Pt(NC_6_F_5_)_2_][SbF_6_]_2_ (**294**) as chiral catalyst [110]. An optimization study revealed that the enantioselectivity varied with the polarity of the solvent. The optimization study disclosed that the enantioselectivity increases with the decrease of the polarity of the solvent (Scheme 68).

**Scheme 69:**
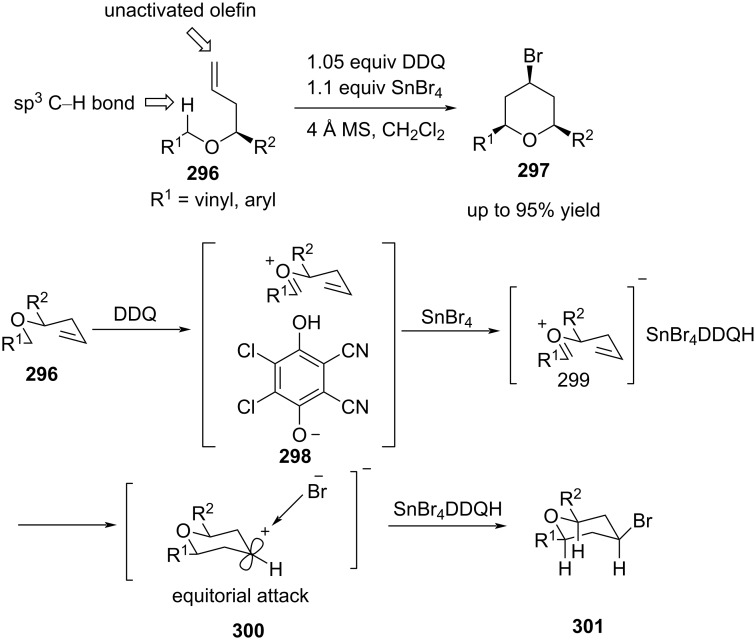
Yu and co-workers’ DDQ-catalyzed asymmetric Prins cyclization strategy to trisubstituted THPs.

Yu and co-workers reported a novel segment-coupling Prins cyclization involving sequential benzylic/allylic C–H bond activation via DDQ oxidation, followed by nucleophilic attack of an unactivated olefin to obtain all-*cis*-trisubstituted Prins products with high stereochemical precision [111]. A single-electron transfer (SET) mechanism was proposed for the above transformation (Scheme 69). A SET from an arene or alkene to DDQ and the subsequent abstraction of hydride from the benzylic or allylic position generated a charge-transfer complex **298**. The complex **298** formed a tin-containing ate oxocarbenium ion complex **299** with SnBr_4_, and then rapid C–C bond formation took place to generate the cyclic intermediate **300**. The subsequent trapping of the carbocation with the bromide ion led to all-*cis*-2,4,6-trisubstituted tetrahydropyran **297** (Scheme 69).

**Scheme 70:**
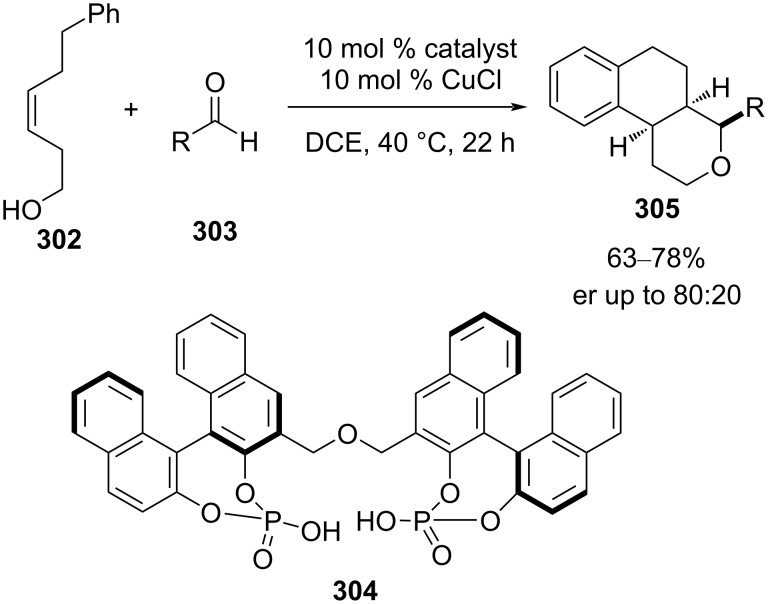
Lalli and Weghe’s chiral-Brønsted-acid- and achiral-Lewis-acid-promoted asymmetric Prins cyclization strategy.

Lalli and van de Weghe reported a chiral BINOL-derived bisphosphoric acid- and CuCl-catalyzed enantioselective tandem Prins–Friedel–Crafts cyclization between homoallylic alcohol **302** and substituted aromatic aldehydes **303** to form hexahydro-1*H*-benzo[*f*]isochromenes **305** with three new contiguous stereocenters in high enantio- and diastereoselectivity [112]. The three new contiguous stereogenic centers formed resulted from an attack of the alkene to the *Si*-face of the oxocarbenium ion, which was followed by a completely diastereoselective Friedel–Crafts reaction (Scheme 70).

**Scheme 71:**
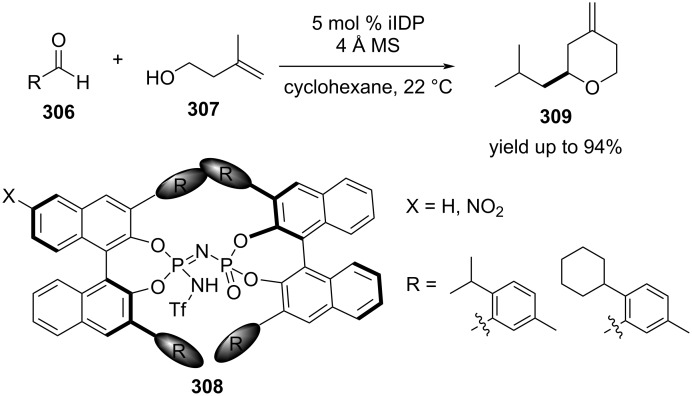
List and co-workers’ iIDP Brønsted acid-promoted asymmetric Prins cyclization strategy.

List and co-workers devised a strategy employing highly acidic confined iminoimidodiphosphate (iIDP) Brønsted acids **308** that catalyzed asymmetric Prins cyclizations of both aliphatic and aromatic aldehydes with alcohol **307** to obtain **309** (Scheme 71) [113]. The introduction of electron-withdrawing nitro groups on the BINOL backbone in the catalysts significantly enhanced the reactivity and enantioselectivity.

**Scheme 72:**
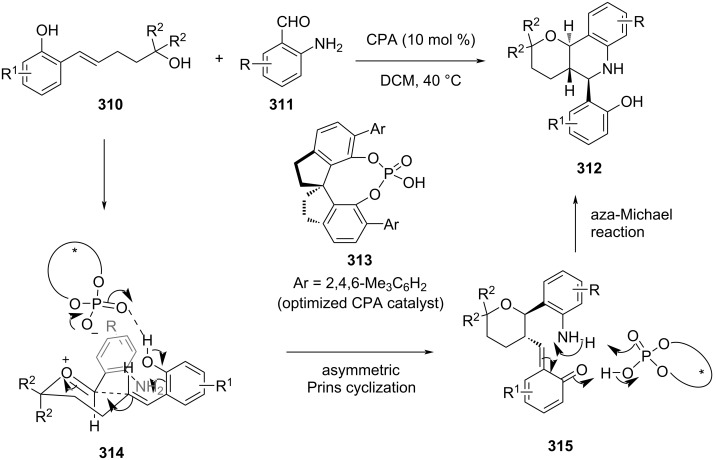
Zhou and co-workers’ strategy for chiral phosphoric acid (CPA)-catalyzed cascade Prins cyclization.

Zhou et al. reported an asymmetric Prins cyclization of in situ-generated quinone methides from phenol-tethered alkenyl alcohol **310** and *o*-aminobenzaldehyde **311** using chiral phosphoric acids (Scheme 72) [114]. Diverse functionalized *trans*-fused pyranotetrahydroquinoline derivatives **312** were synthesized in excellent yield and selectivity (up to 99% yield and 99% ee).

**Scheme 73:**
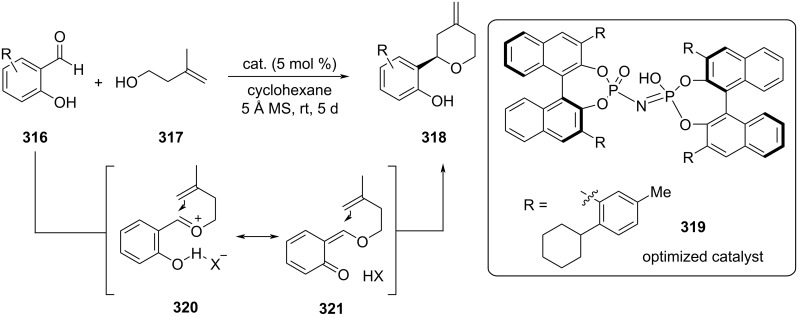
List and co-workers’ approach for asymmetric Prins cyclization using chiral imidodiphosphoric acid **319**.

List et al. reported a chiral imidodiphosphoric acid-catalyzed asymmetric Prins cyclization with salicylaldehyde **316** and 3-methylbut-3-en-1-ol (**317**) to afford 4-methylenetetrahydropyrans **318** with high enantioselectivity (Scheme 73) [115]. A chiral bis-BINOL-based imidophosphoric acid **319** was efficient in this reaction, and the extreme bulkiness of this catalyst was the key to a successful transformation. This reaction proceeded via a Prins cyclization mechanism, activated by chiral acid **319**.

## Conclusion

Prins cyclization strategies have been proven as a reliable and robust method for the stereoselective construction of THP rings. Many of these strategies have been utilized for the elegant synthesis of natural products. In this review, we portrayed an inspection of twenty years in the arena of the development of Prins cyclizations and the further exploration of these strategies in the total synthesis of natural products. This up-to-date information showcases the knowledge gained in this area. In either case, it is hoped that the challenge of stereoselective construction of THP rings in the context of natural product synthesis will continue to inspire synthetic chemists to develop new methods in the coming years.
